# Hypoxic Reprogramming of ACOX1-Driven HSP90AB1 Crotonylation Stabilizes Thioredoxin to Orchestrate Redox Homeostasis in Oral Squamous Cell Carcinoma

**DOI:** 10.34133/research.1129

**Published:** 2026-02-10

**Authors:** Xiteng Yin, Yuyang Zhang, Yan Zhang, Meng Zhou, Jingwei Zhang, Zhi Wang, Wenguang Xu, Chuanhui Song, Jianchuan Ran, Lin Lin, Xingyu Luo, Wei Han

**Affiliations:** ^1^ Nanjing Stomatological Hospital, Affiliated Hospital of Medical School, Institute of Stomatology, Nanjing University, Nanjing 210008, China.; ^2^ Department of Oral and Maxillofacial Surgery, The Affiliated Stomatological Hospital of Xuzhou Medical University, Xuzhou 221003, China.; ^3^ Key Laboratory of Drug Metabolism and Pharmacokinetics, State Key Laboratory of Natural Medicines, China Pharmaceutical University, Nanjing 210008, China.

## Abstract

Hypoxia promotes oral squamous cell carcinoma (OSCC) progression by disrupting redox equilibrium; however, how tumor cells precisely calibrate prosurvival reactive oxygen species levels remains unclear. This study identifies a hypoxia-inducible signaling axis centered on the posttranslational crotonylation of the molecular chaperone heat shock protein 90 alpha family class B member 1 (HSP90AB1), which stabilizes thioredoxin (TXN) to constrain oxidative stress. Hypoxia triggered the hypoxia-inducible factor-1α (HIF-1α)-dependent transcriptional up-regulation of acyl-CoA oxidase 1 (ACOX1), increasing the level of crotonyl-CoA to drive the site-specific crotonylation of HSP90AB1 at lysine 265 (K265cr). Molecular dynamics simulations revealed that K265 crotonylation induced the conformational compaction of HSP90AB1, strengthening its interaction with TXN and enhancing its stability. This chaperone–client axis effectively buffers reactive oxygen species to protumorigenic thresholds, promoting proliferation and conferring cisplatin resistance. Clinically, HIF-1α/ACOX1/HSP90AB1 K265cr/TXN pathway activation is correlated with advanced disease and reduced survival in OSCC patients. Crucially, the HSP90AB1 K265R mutation or pharmacological inhibition of ACOX1 (10,12-tricosadiynoic acid) or TXN (1-methyl-propyl 2-imidazolyl disulfide, PX-12) synergizes with cisplatin to suppress tumor growth in vivo by disrupting redox adaptation. These findings reveal that crotonylation is a hypoxia-sensitive rheostat for TXN-mediated redox control, suggesting that the ACOX1–HSP90AB1–TXN axis is a therapeutic vulnerability in therapy-resistant OSCC.

## Introduction

Oral squamous cell carcinoma (OSCC) is an aggressive malignancy notorious for its dysregulated redox homeostasis, which is a fundamental driver of tumor initiation, progression, and therapeutic resistance [1,2]. Within this pathological landscape, reactive oxygen species (ROS) play a complex dualistic role: at moderate levels, they function as potent signaling molecules that activate protumorigenic pathways and facilitate malignant progression; however, excessive ROS accumulation triggers irreversible oxidative damage and cell death [3–5]. Tumor cells must therefore precisely calibrate ROS levels through sophisticated antioxidant systems [6–8]; however, the molecular mechanisms enabling this equilibrium in OSCC remain incompletely understood.

The thioredoxin (TXN) system forms a cornerstone of the cellular redox regulatory machinery [9]. TXN, a pivotal redox-active chaperone, sustains intracellular reducing power and safeguards against oxidative stress [10]. Its overexpression is a recurring feature across diverse malignancies and is strongly correlated with aggressive phenotypes and poor patient prognosis [11–13]. Nevertheless, the upstream mechanisms governing TXN regulation in OSCC, particularly under pathophysiological stressors such as pervasive tumor hypoxia, are poorly defined.

Hypoxia, a defining hallmark of the OSCC microenvironment, orchestrates profound adaptive responses primarily through the stabilization and activation of hypoxia-inducible factor-1α (HIF-1α) [14–16]. This master transcriptional regulator reprograms cellular metabolism and induces unique posttranslational modifications (PTMs) that dynamically modulate protein function, stability, and interactions [17–19]. Among the emerging repertoire of metabolism-sensitive PTMs, lysine crotonylation (Kcr) has garnered considerable interest for its regulatory roles in chromatin dynamics and diverse cellular processes [20,21]. However, its functional impact on core redox regulators such as TXN and its potential integration into hypoxic signaling networks in cancer represent a marked knowledge gap.

Our comprehensive crotonylome analysis conducted specifically under hypoxic conditions revealed marked global up-regulation of nonhistone Kcr modifications in OSCC cells. Strikingly, crotonylation at lysine 265 of heat shock protein 90 alpha family class B member 1 (HSP90AB1 K265cr) has emerged as the most dynamic hypoxia-responsive site [22]. HSP90AB1, a central molecular chaperone and stress-response protein, plays a critical role in stabilizing a vast array of client proteins, including numerous oncoproteins and signaling molecules [23–25]. Its function is intricately modulated by PTMs, which dictate its ATPase cycle, cochaperone binding, and client protein interactions [26]. While canonical PTMs such as acetylation and phosphorylation have been extensively studied for their roles in fine-tuning heat shock protein 90 (HSP90) chaperone activity in cancer, crotonylation remains a relatively unexplored regulatory layer in chaperone biology. These findings position HSP90AB1, particularly its hypoxia-induced crotonylation modification, as a compelling mechanistic nexus potentially linking the hypoxic tumor microenvironment (TME) to the rewiring of redox homeostasis, which is essential for cancer cell survival and adaptation.

In this study, we identified a novel hypoxia-responsive signaling axis essential for maintaining protumorigenic ROS homeostasis in OSCC. We demonstrated that HIF-1α transcriptionally up-regulates acyl-CoA oxidase 1 (ACOX1), a key enzyme in fatty acid β-oxidation, leading to the specific crotonylation of HSP90AB1 at K265. This modification induces a conformational change in HSP90AB1 that enhances its binding affinity for TXN, resulting in TXN stabilization and increased antioxidant capacity. Clinically, the activation of this HIF-1α/ACOX1/HSP90AB1 K265cr/TXN axis is correlated with advanced disease and poor prognosis in OSCC patients. Importantly, targeted disruption of this axis sensitizes OSCC tumors to ROS-inducing therapeutics such as cisplatin, revealing its therapeutic potential.

## Results

### Elevated TXN protein expression, but not mRNA expression, is associated with tumor progression and poor prognosis in OSCC

**Fig. 1.**
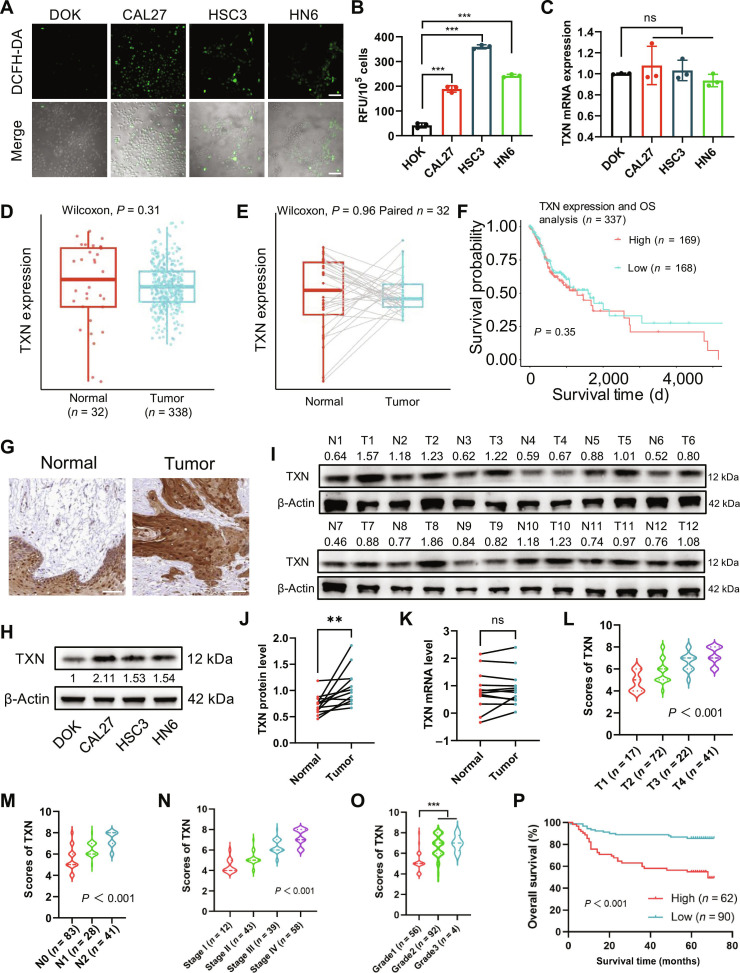
Differential expression of thioredoxin (TXN) protein and messenger RNA (mRNA) in oral squamous cell carcinoma (OSCC). (A) Representative confocal microscopy images showing intracellular reactive oxygen species (ROS) levels detected by 2′,7′-dichlorodihydrofluorescein diacetate (DCFH-DA) staining in dysplastic oral keratinocyte (DOK) and OSCC cell lines (CAL27, HSC3, and HN6) (200×, scale bars, 100 μm). (B) Quantification of DCFH-DA fluorescence intensity in OSCC cell lines (CAL27, HSC3, and HN6) versus that in DOK cells (RFU, relative fluorescence units) (***P < 0.001, one-way analysis of variance [ANOVA]). (C) Quantitative real-time polymerase chain reaction (qPCR) analysis showing comparable *TXN* mRNA expression across cell lines (ns, not significant, one-way ANOVA). (D) *TXN* mRNA expression in unpaired OSCC tumor tissues (*n* = 338) versus normal oral tissues (*n* = 32) from The Cancer Genome Atlas (TCGA). (E) *TXN* mRNA expression in paired OSCC tumor tissues and adjacent normal tissues (*n* = 32 pairs) from TCGA. (F) Kaplan–Meier overall survival (OS) analysis of patients in the TCGA OSCC cohort stratified by the *TXN* mRNA expression level. (G) Representative immunohistochemistry (IHC) images of the TXN protein in human OSCC tumor tissue and normal oral mucosa from the Human Protein Atlas (200×, scale bars, 100 μm). (H) Western blot analysis of TXN protein expression in DOK and OSCC cell lines (CAL27, HSC3, and HN6). β-Actin served as a loading control. (I) Western blots and (J) quantitative analysis of the TXN protein in 12 paired clinical OSCC samples (T, tumor; N, adjacent normal; **P < 0.01, paired *t* test). (K) qPCR analysis of TXN mRNA in paired clinical samples (ns, not significant, paired *t* test). (L to O) IHC analysis of the TXN protein in 152 OSCC patients: associations with (L) T stage, (M) N stage, (N) pathological stage, and (O) histological grade (***P < 0.001, one-way ANOVA). (P) Kaplan–Meier overall survival curves for 152 OSCC patients stratified by the TXN protein expression level (log-rank test).

To elucidate the mechanisms underlying redox homeostasis in OSCC, given the dual role of moderate ROS levels in promoting tumor progression versus the cytotoxicity of excessive ROS, we assessed the redox state and the key antioxidant protein TXN. Notably, OSCC cell lines (CAL27, HSC3, and HN6) presented significantly higher basal intracellular ROS levels than nontumorigenic oral keratinocytes (dysplastic oral keratinocytes [DOKs]), as quantified by 2′,7′-dichlorodihydrofluorescein diacetate (DCFH-DA) fluorescence (Fig. 1A and B). This elevation in ROS, presumably under precise control, prompted us to investigate the TXN system. Surprisingly, *TXN* messenger RNA (mRNA) levels did not significantly differ across the OSCC cell lines (Fig. 1C). Consistently, analysis of The Cancer Genome Atlas (TCGA) database revealed no differential *TXN* mRNA expression between OSCC tumors and normal tissues in bulk (Fig. 1D) or paired samples (Fig. 1E). Moreover, *TXN* mRNA expression correlated with neither clinicopathological parameters (T/N stage, pathological stage/grade; Fig. S1A to D) nor overall survival (Fig. 1F), suggesting limited functional relevance at the transcriptional level.

In contrast, the Human Protein Atlas revealed elevated TXN protein levels in OSCC tumors (Fig. 1G). This mRNA–protein interaction was experimentally validated, and Western blotting confirmed that the TXN protein was up-regulated in all OSCC lines compared with that in DOKs (Fig. 1H). Critically, analysis of 12 paired clinical samples revealed significantly higher TXN protein levels in tumors (Fig. 1I and J), despite unaltered mRNA levels (Fig. 1K), which aligns with public data. Immunohistochemistry (IHC) of an independent OSCC cohort revealed that a high TXN protein expression was associated with an advanced T stage (Fig. 1L), nodal metastasis (Fig. 1M), a high pathological stage (Fig. 1N), and a high grade (Fig. 1O). Most importantly, patients with high TXN protein levels exhibited significantly worse overall survival (Fig. 1P). Collectively, these results demonstrate that elevated TXN protein, which is uncoupled from mRNA expression, serves as a clinically important biomarker of tumor aggressiveness and poor prognosis in OSCC. This posttranscriptional dysregulation suggests a microenvironment-driven mechanism enabling redox adaptation in malignant cells, independent of genetic alterations at the TXN locus.

### Hypoxia induces dynamic ROS flux and posttranscriptional TXN up-regulation in OSCC

**Fig. 2.**
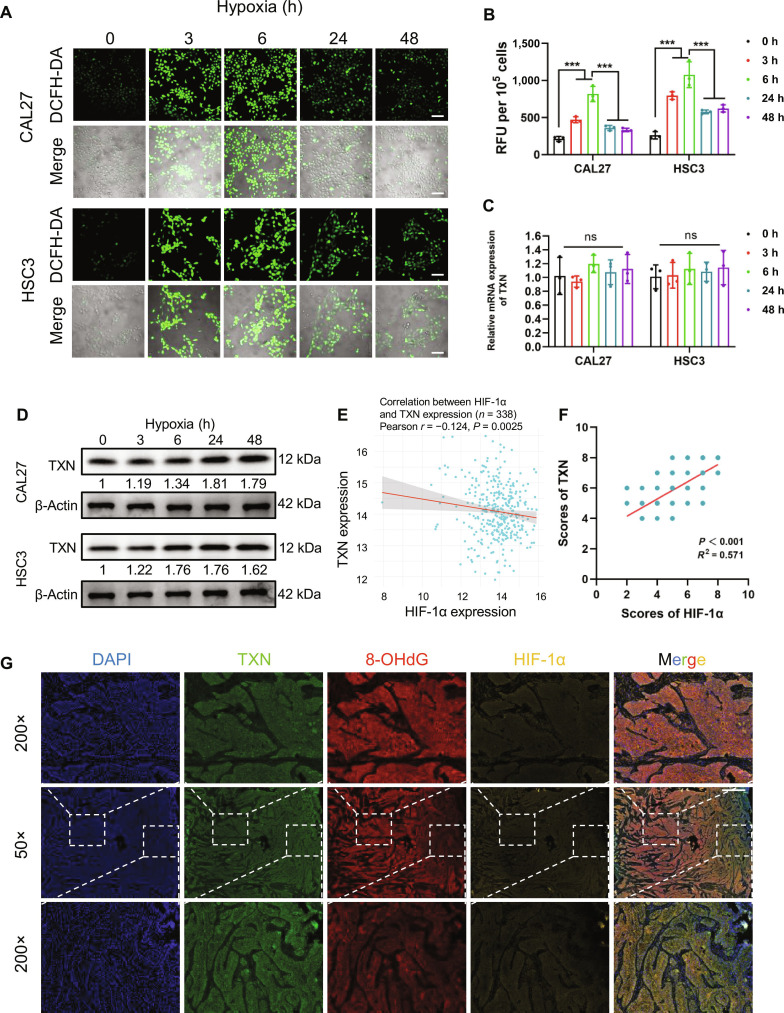
Hypoxia induces dynamic ROS flux and posttranscriptional TXN up-regulation in OSCC. (A) Representative confocal microscopy images of DCFH-DA-stained CAL27 and HSC3 cells under hypoxic conditions at the indicated time points (h) (200×, scale bars, 100 μm). (B) Quantification of DCFH-DA fluorescence intensity in CAL27 and HSC3 cells during hypoxia exposure (***P < 0.001, one-way ANOVA). (C) qPCR analysis of the *TXN* mRNA levels in CAL27 and HSC3 cells during hypoxia exposure (ns, not significant, one-way ANOVA). (D) Western blot analysis of TXN protein expression in CAL27 and HSC3 cells under hypoxia for the indicated durations (h). β-Actin served as a loading control. (E) Scatter plot of *HIF-1α* versus *TXN* mRNA expression levels in OSCC samples from the TCGA cohort (*n* = 338, Pearson correlation). (F) Scatter plot of hypoxia-inducible factor-1α (HIF-1α) versus TXN protein expression levels in 152 OSCC patients (*n* = 152, Pearson correlation). (G) Multiplex immunohistochemistry (mIHC) of TXN (green), 8-hydroxy-2′-deoxyguanosine (8-OHdG; red), and HIF-1α (yellow) in clinical OSCC sections. Nuclei were counterstained with 4′,6-diamidino-2-phenylindole (DAPI; blue) (scale bars, 100 μm).

Hypoxia is a hallmark of solid tumors that perturbs redox balance by both generating ROS and inducing antioxidant adaptations [27,28]. Given our discovery of TXN protein up-regulation and its clinical relevance, we hypothesized that the hypoxic TME orchestrates posttranscriptional TXN regulation to maintain protumorigenic ROS levels. To test this hypothesis, we investigated the dynamic interplay between hypoxia-induced ROS flux and TXN expression in OSCC models. Time-course analysis under hypoxia revealed a biphasic ROS response in OSCC lines: acute hypoxia (3 to 6 h) induced rapid ROS accumulation, whereas prolonged exposure (24 to 48 h) caused a partial reduction, although the levels remained elevated compared with those under normoxia (Fig. 2A and B and Fig. S2A and B). This adaptation suggested the activation of redox regulatory mechanisms.

Notably, hypoxia induced progressive TXN protein up-regulation without altering *TXN* mRNA (Fig. 2C and D and Fig. S2C and D), reinforcing posttranscriptional control and linking it directly to the hypoxic microenvironment. While TCGA data revealed no correlation between *HIF-1α* and *TXN* mRNA in OSCC (Fig. 2E), IHC analysis confirmed a robust positive association at the protein level (Fig. 2F), underscoring the functional interplay between hypoxia signaling and TXN regulation. Strikingly, multiplex IHC analysis of OSCC sections revealed that areas with high TXN expression coincided with elevated HIF-1α levels and notably reduced expression of the oxidative damage marker 8-hydroxy-2′-deoxyguanosine (8-OHdG). These findings establish that hypoxia orchestrates TXN protein up-regulation through posttranscriptional mechanisms, enabling the adaptation of ROS, which is essential for OSCC progression. However, the upstream signaling events coordinating redox sensing with antioxidant effector systems such as TXN remain incompletely defined. PTMs represent a critical regulatory layer for cellular adaptation to microenvironmental stressors such as hypoxia [29,30].

### Hypoxia induces site-specific crotonylation of HSP90AB1 at K265 to modulate ROS homeostasis

**Fig. 3.**
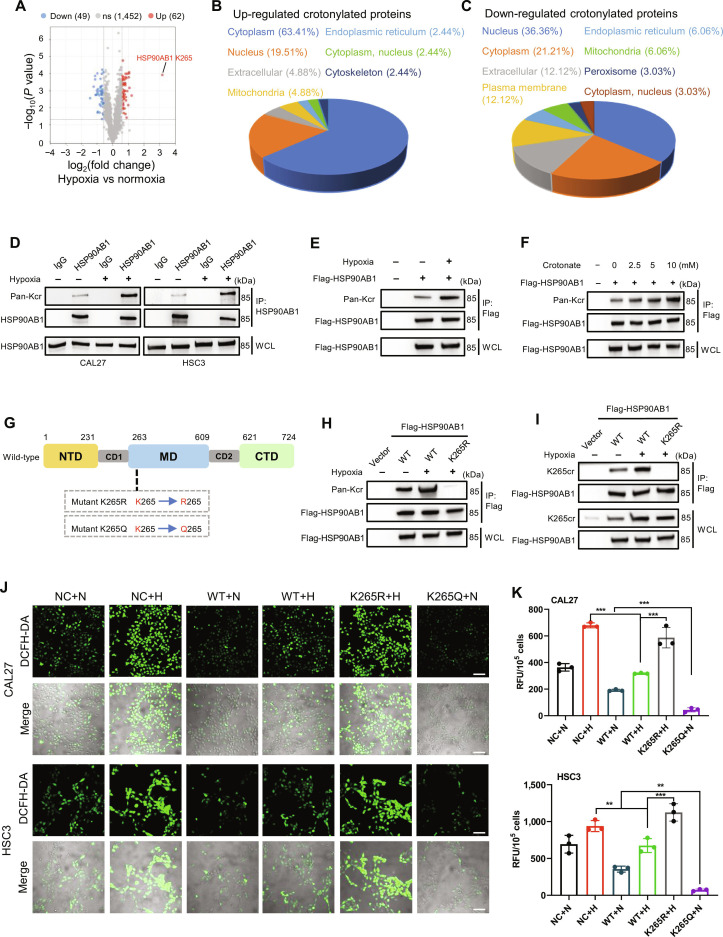
Hypoxia induces the site-specific crotonylation of heat shock protein 90 alpha family class B member 1 (HSP90AB1) at lysine 265 (K265) to regulate redox homeostasis in OSCC. (A) Volcano plot of crotonylation sites in CAL27 cells after 24 h of hypoxia versus normoxia. Sites are colored red for fold changes >1.5 and *P* < 0.05 and blue for fold changes <−1.5 and *P* < 0.05. (B and C) Subcellular localization analysis of (B) up-regulated and (C) down-regulated crotonylated proteins. (D) Immunoprecipitation (IP) of endogenous HSP90AB1 in CAL27/HSC3 cells under normoxia/hypoxia (24 h) and probing with a pan-Kcr antibody (IgG, immunoglobulin G; WCL, whole-cell lysate). (E) IP of Flag-HSP90AB1 in HEK293T cells under normoxia and hypoxia probed with a pan-Kcr antibody. (F) Crotonylation levels of Flag-HSP90AB1 in HEK293T cells treated with sodium crotonate (0 to 10 mM) under normoxia (24 h). (G) Domain structure of HSP90AB1 (NTD, N-terminal domain; MD, middle domain; CD, charged domain; CTD, C-terminal domain) and K265 mutation schematic (K, lysine; R, arginine; Q, glutamine). (H and I) IP of Flag-HSP90AB1 in HEK293T cells reconstituted with HSP90AB1 variants (wild type [WT], K265R, and K265Q) under normoxia/hypoxia (24 h) and probed with (H) pan-Kcr and (I) site-specific K265cr antibodies. (J) Representative confocal images of DCFH-DA staining in HSP90AB1-knockdown CAL27/HSC3 cells reconstituted with HSP90AB1 variants under normoxia/hypoxia (24 h) (200×, scale bars, 100 μm). NC, negative control; N, normoxia; H, hypoxia. (K) Quantification of DCFH-DA fluorescence intensity in HSP90AB1-knockdown CAL27/HSC3 cells reconstituted with HSP90AB1 variants under normoxia/hypoxia (24 h) (**P < 0.01 and ***P < 0.001, one-way ANOVA).

Protein crotonylation, a dynamic PTM increasingly implicated in tumorigenesis, results in aberrant profiles in malignancies, including hepatocellular carcinoma, colorectal cancer, and glioblastoma [31–33]. Building upon this expanding oncogenic context, our prior work identified hypoxia-induced alterations in the OSCC crotonylome, with HSP90AB1 K265 crotonylation (K265cr) exhibiting the most pronounced up-regulation (Fig. 3A). Hypoxia-induced crotonylation changes displayed spatial specificity, predominantly affecting nonhistone cytoplasmic proteins (up-regulated) rather than nuclear histones (down-regulated) (Fig. 3B and C).

HSP90AB1, a member of the HSP90 family, plays a pivotal role in various cellular processes [24]. To validate the crotonylome results, we performed immunoprecipitation (IP) assays followed by immunoblotting with an antibody against pan-crotonyl lysine (pan-Kcr). Consistent with our previous findings, hypoxic treatment led to a significant increase in HSP90AB1 Kcr levels in both CAL27 and HSC3 cells (Fig. 3D). Similarly, hypoxia-induced up-regulation of HSP90AB1 Kcr was also observed in HEK293T cells (Fig. 3E). Given that crotonate serves as the precursor for protein crotonylation [34], we further examined its role in promoting the HSP90AB1 Kcr. Notably, crotonate supplementation was sufficient to increase HSP90AB1 Kcr levels even under normoxic conditions (Fig. 3F), highlighting the critical role of the substrate in driving this PTM.

K265 resides within the HSP90AB1 middle domain, which is crucial for client protein interactions, and its surrounding sequence is highly conserved (Fig. S3A). This strong evolutionary conservation suggests that the K265 site and its flanking residues likely play a critical functional or regulatory role. To determine whether lysine 265 serves as the principal crotonylation site of HSP90AB1 under hypoxic conditions, site-directed mutagenesis was performed. Specifically, the 265th amino acid of HSP90AB1 was mutated to arginine (K265R), which eliminates crotonylation at this site, or to glutamine (K265Q), which mimics the functional properties of crotonylation (Fig. 3G). IP analysis of HEK293T cells expressing Flag-tagged constructs confirmed that K265 was the primary hypoxia-induced crotonylation site, as the K265R mutation abolished the hypoxia-induced increase in Kcr (Fig. 3H). A custom HSP90AB1 K265cr antibody was generated, and its specificity was rigorously validated with these mutants (Fig. 3I).

Lentiviral short hairpin RNA (shRNA)-mediated *HSP90AB1* knockdown in OSCC cells (Fig. S3B and C) enabled functional rescue studies. Reintroduction of wild-type (WT) HSP90AB1 restored hypoxia-induced K265cr, whereas the K265R and K265Q mutants failed to show K265cr under any condition, confirming site specificity (Fig. S3D and E). Functional analysis revealed that K265cr regulates ROS homeostasis: compared with WT cells, hypoxia-exposed K265R-expressing cells presented significantly elevated ROS levels, whereas K265Q-expressing cells presented reduced basal ROS even under normoxia (Fig. 3J and K). Given that cisplatin exerts its cytotoxic effects largely through the induction of ROS, we next investigated whether K265cr-dependent ROS modulation influences cellular sensitivity to this chemotherapeutic agent. In functional assays (Cell Counting Kit-8 [CCK-8], 5-ethynyl-2′-deoxyuridine [EdU] incorporation, and colony formation) performed under cisplatin treatment, hypoxia was found to induce significant cisplatin resistance in cells expressing wild-type HSP90AB1 (K265 WT). This resistant phenotype was effectively reversed by the K265R mutation, which restored cisplatin sensitivity even under hypoxic conditions. Conversely, the crotonylation-mimetic K265Q mutation was sufficient to confer cisplatin resistance under normoxia, mirroring the hypoxic effect observed with the WT protein (Fig. S3F to J). These findings establish hypoxia-induced HSP90AB1 K265cr as a redox regulator that modulates ROS homeostasis and chemosensitivity in OSCC. While this PTM-mediated functional switch implicates HSP90AB1 as a hypoxic stress sensor, the mechanism underlying its antioxidant regulation remains unresolved.

### Chaperone HSP90AB1 stabilizes TXN to maintain redox homeostasis in OSCC

**Fig. 4.**
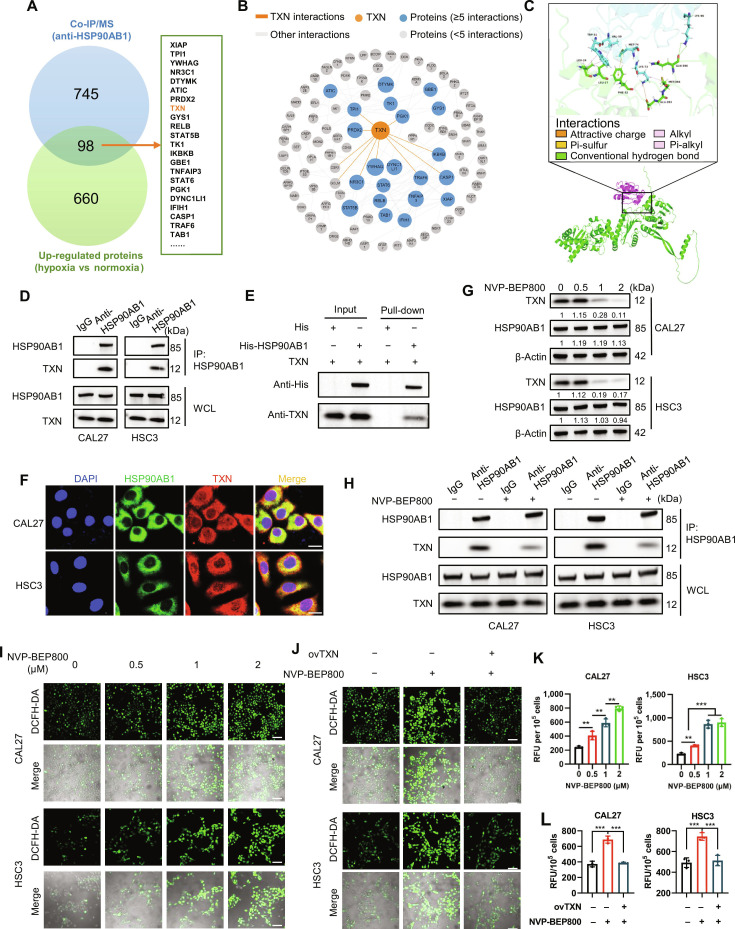
Experimental analysis of the HSP90AB1–TXN interaction and functional relationships. (A) Venn diagram comparing the hypoxia-up-regulated proteome (758 proteins, green) and the HSP90AB1 interactome (843 proteins, blue) identified by co-IP/mass spectrometry (MS), showing 98 overlapping candidates. (B) Protein–protein interaction network of the 98 candidates from panel (A). (C) Structural models of the HSP90AB1–TXN complex: (bottom) docked conformation (HSP90AB1, green; TXN, purple); (top) 3-dimensional interaction diagram of interface residues (HSP90AB1, green; TXN, cyan). (D) Endogenous co-IP of HSP90AB1 and TXN in CAL27/HSC3 cells. IgG: negative control. (E) In vitro pull-down assay of His-HSP90AB1 with TXN. (F) Immunofluorescence (IF) staining of HSP90AB1 (red) and TXN (green) in CAL27/HSC3 cells. Nuclei counterstained with DAPI (blue) (600×, scale bars, 20 μm). (G) Western blot analysis of the indicated proteins in CAL27/HSC3 cells treated with NVP-BEP800 (0 to 2 μM, 24 h). (H) Co-IP of HSP90AB1 and TXN in CAL27/HSC3 cells following NVP-BEP800 treatment (2 μM). (I) Representative confocal microscopy images of DCFH-DA staining in CAL27/HSC3 cells treated with NVP-BEP800 (0 to 2 μM, 24 h) (200×, scale bars, 100 μm). (J) Representative confocal microscopy images of DCFH-DA-stained CAL27/HSC3 cells with or without TXN overexpression and NVP-BEP800 treatment (200×, scale bars, 100 μm). ovTXN, TXN overexpression. (K) Quantification of DCFH-DA fluorescence intensity in CAL27/HSC3 cells treated with NVP-BEP800 (0 to 2 μM, 24 h) (**P < 0.01 and ***P < 0.001, one-way ANOVA). (L) Quantification of DCFH-DA fluorescence intensity in CAL27/HSC3 cells with or without TXN overexpression and NVP-BEP800 treatment (2 μM) (***P < 0.001, one-way ANOVA).

As a molecular chaperone, HSP90AB1 critically depends on protein–protein interactions to fulfill its regulatory functions. To elucidate the mechanism underlying the HSP90AB1 K265cr-mediated regulation of ROS homeostasis, we performed IP coupled with mass spectrometry (MS) to identify HSP90AB1 interactors (Fig. S4A and B). Given that HSP90AB1 binding typically stabilizes client proteins, we conducted a quantitative proteomic analysis comparing CAL27 cells under normoxic and hypoxic conditions. This identified hypoxia-up-regulated proteins, which were cross-referenced with the HSP90AB1 interactome to yield 98 overlapping candidates (Fig. 4A). Subsequent protein–protein interaction network analysis via the Search Tool for the Retrieval of Interacting Genes/Proteins (STRING) database prioritized TXN as a key component within the HSP90AB1 signaling axis (Fig. 4B).

Protein–protein docking analysis confirmed the predicted interaction with high confidence (docking score: −607.83; confidence score: 0.9999). The molecular model localized the binding interface primarily to residues 393 to 396 within the client-binding domain of HSP90AB1 (Fig. 4C). Physiological validation was achieved through co-IP, which revealed an endogenous HSP90AB1–TXN interaction in both CAL27 and HSC3 cells, with no binding observed for control immunoglobulin G (IgG) (Fig. 4D and Fig. S4C). In vitro His pull-down assays using purified His-tagged HSP90AB1 and lysates from TXN-overexpressing HEK293T cells established direct binding (Fig. 4E). Furthermore, immunofluorescence co-staining confirmed strong cytoplasmic colocalization (Fig. 4F). Collectively, these findings demonstrate a specific, direct interaction between HSP90AB1 and TXN, suggesting a regulatory axis for ROS homeostasis.

Functional interrogation revealed that HSP90AB1 regulates TXN posttranslationally. *HSP90AB1* knockdown significantly increased TXN protein levels without altering *TXN* mRNA expression in CAL27 and HSC3 cells (Fig. S4D and E). Similarly, pharmacological inhibition of HSP90AB1 via the adenosine triphosphate (ATP)-competitive inhibitor NVP-BEP800 down-regulated TXN protein expression (Fig. 4G). Subsequent cycloheximide (CHX) chase assays confirmed the presence of HSP90AB1-dependent TXN stability (Fig. S4F). To delineate the specific degradation route, CHX chase assays were performed in the presence of a proteasome inhibitor (MG132) or a lysosome inhibitor (chloroquine). In both CAL27 and HSC3 cells, MG132, but not chloroquine, effectively rescued the accelerated TXN degradation induced by NVP-BEP800, indicating that TXN is predominantly degraded via the proteasomal pathway upon HSP90AB1 inhibition (Fig. S4G). Furthermore, NVP-BEP800 treatment markedly reduced HSP90AB1–TXN binding (Fig. 4H). Consistently, NVP-BEP800-induced ROS accumulation was rescued by TXN overexpression (Fig. 4I to L). These data establish a chaperone–client axis in which HSP90AB1 posttranslationally stabilizes TXN to regulate redox homeostasis. However, how hypoxia-induced K265 crotonylation mechanistically enhances the chaperone activity of HSP90AB1 toward TXN remains structurally undefined.

### Crotonylation at K265 enhances HSP90AB1-mediated TXN stabilization

**Fig. 5.**
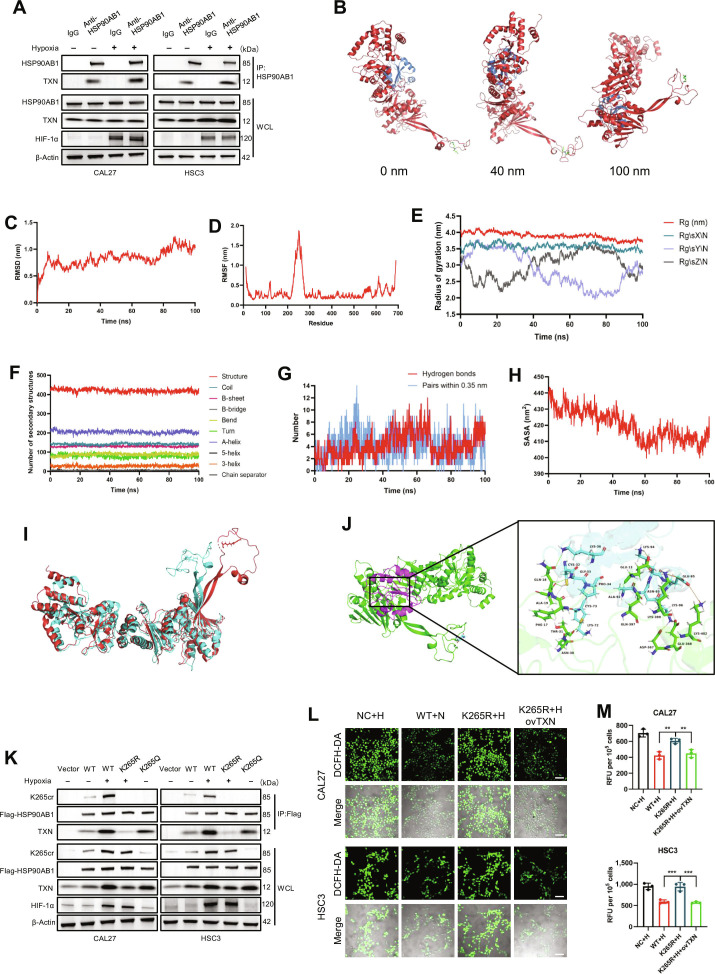
K265 crotonylation induces conformational remodeling of HSP90AB1 to enhance TXN binding and stabilization. (A) Co-IP of endogenous HSP90AB1–TXN complexes in CAL27/HSC3 cells under normoxia or hypoxia. (B) Molecular dynamics simulation snapshots (100 ns) of the HSP90AB1–TXN complex: HSP90AB1 with K265 crotonylation (red), TXN (blue), and the crotonyl moiety at K265 (green). (C) Root mean square deviation (RMSD) trajectory of the HSP90AB1–TXN complex during molecular dynamics simulation. (D) Root mean square fluctuation (RMSF) per residue analysis of HSP90AB1 during simulation. (E) Radius of gyration measurements of HSP90AB1 during simulation. (F) Secondary structure composition analysis of HSP90AB1 (Define Secondary Structure of Proteins [DSSP] method). (G) Hydrogen bond formation between HSP90AB1 and TXN with simulation time. (H) Solvent-accessible surface area (SASA) measurements of the HSP90AB1–TXN complex during simulation. (I) Structural superposition of the initial (red) and equilibrated (cyan) HSP90AB1 conformations. (J) Molecular docking model of K265cr-modified HSP90AB1 (green) with TXN (purple/cyan). (K) Co-IP of Flag-HSP90AB1 in HSP90AB1-knockdown CAL27/HSC3 cells reconstituted with HSP90AB1 variants under normoxia/hypoxia (24 h). (L) Representative confocal microscopy images of DCFH-DA-stained CAL27/HSC3 cells expressing HSP90AB1 variants with/without TXN overexpression under normoxia/hypoxia (24 h) (200×, scale bars, 100 μm). (M) Quantification of DCFH-DA fluorescence intensity in CAL27/HSC3 cells expressing HSP90AB1 variants with/without TXN overexpression under normoxia/hypoxia (24 h) (**P < 0.01 and ***P < 0.001, one-way ANOVA).

Hypoxic treatment significantly increased TXN binding to HSP90AB1 (Fig. 5A), indicating that K265 site-specific crotonylation potentiates the HSP90AB1–TXN protein interaction. To mechanistically validate the structural impact of K265 crotonylation on the conformational dynamics of HSP90AB1, we conducted molecular dynamics simulations. After the introduction of a crotonyl group at the K265 site, substantial spatial conformational changes were observed in the local structure of HSP90AB1 (Fig. 5B). During the simulation, the root mean square deviation of the HSP90AB1–TXN complex fluctuated between 70 and 85 ns before stabilizing, suggesting conformational rearrangements near the modified site (Fig. 5C). Decomposition analysis revealed that these dynamic fluctuations originated predominantly from HSP90AB1, whereas TXN maintained relative conformational stability throughout the simulation timeframe (Fig. S5A and B). Additionally, root mean square fluctuation (RMSF) analyses highlighted marked flexibility in the region encompassing amino acids 220 to 270 of HSP90AB1, with a maximum fluctuation of ~1.87, underscoring its role as a dynamic and modifiable region (Fig. 5D). Concomitantly, the RMSF values of the TXN exhibit small fluctuations and are relatively stable (Fig. S5C). The radius of gyration (Rg), a critical parameter describing the spatial distribution of molecular mass, is employed to evaluate conformational changes and temporal variations in molecular compactness during simulations. As shown in Fig. 5E, the progressive decrease in Rg values for HSP90AB1 throughout the simulation trajectory indicates structural compaction. In contrast, TXN maintained relatively stable Rg values, demonstrating minimal variation in its spatial extent during the simulation period (Fig. S5D). Define Secondary Structure of Proteins (DSSP) analysis revealed that the secondary structure content of both HSP90AB1 and TXN remained relatively stable throughout the molecular dynamics simulation (Fig. 5F and Fig. S5E to G). Crucially, postsimulation analysis revealed a marked increase in intermolecular hydrogen bond formation between HSP90AB1 and TXN, suggesting substantial strengthening of their interaction interface during the trajectory (Fig. 5G). Notably, the solvent-accessible surface area (SASA) of the HSP90AB1–TXN complex progressively decreased during the simulation, contracting from 441.10 to 412.05 nm^2^ (Fig. 5H). This pronounced contraction (ΔSASA = 29.05 nm^2^) demonstrated enhanced interfacial complementarity between the binding partners, which was driven primarily by structural reorganization of HSP90AB1 (from 397.489 to 361.653 nm^2^), which reduced its solvent-exposed hydrophobic patches and improved its geometric complementarity with TXN (Fig. S5H and I). The conformational changes in HSP90AB1 before and after simulation are shown in Fig. 5I. These results suggest that K265cr induces conformational compression in HSP90AB1, increasing its interfacial complementarity with TXN. Notably, molecular docking simulations of K265-site crotonylation-modified HSP90AB1 with TXN revealed a significantly enhanced interaction profile compared with that of unmodified HSP90AB1, with a docking score of −640.24 and a confidence score of 0.9999 (Fig. 5J).

To validate these results, CAL27 and HSC3 cells were transfected with HSP90AB1 variants and subjected to normoxic or hypoxic conditions. As shown in Fig. 5K, hypoxia exposure induced significant TXN protein up-regulation and increased the binding affinity of HSP90AB1–TXN in WT-expressing cells. In contrast, the K265R mutation abolished hypoxia-responsive regulation, resulting in no significant increase in TXN expression or minimal binding capacity. Strikingly, cells expressing the K265Q mutant plasmid exhibited hypoxia-independent TXN up-regulation and sustained strong HSP90AB1–TXN interactions. K265 enhances the stability of the TXN protein (Fig. S5J). These findings collectively demonstrate that HSP90AB1 K265 crotonylation enhances its capacity to stabilize the TXN protein by modulating protein–protein interactions and altering the conformational dynamics of its client-binding domain. Critically, TXN overexpression rescued ROS dysregulation in K265R mutants (Fig. 5L and M).

### HIF-1α up-regulates HSP90AB1 K265cr by promoting the expression of ACOX1

**Fig. 6.**
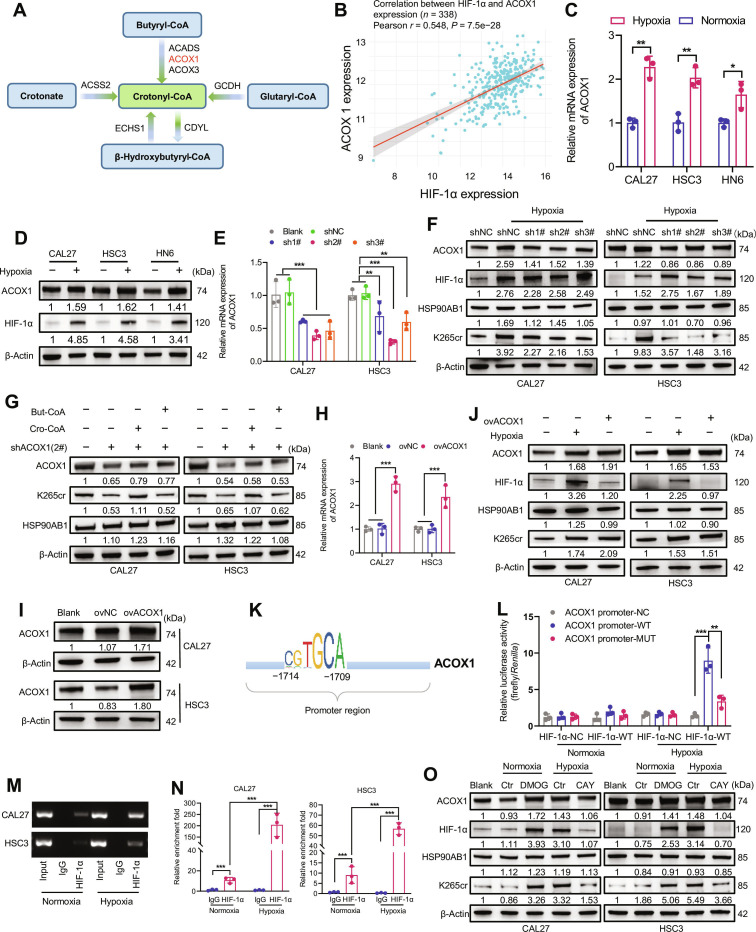
HIF-1α transcriptionally activates acyl-CoA oxidase 1 (ACOX1) to promote HSP90AB1 K265 crotonylation. (A) Schematic diagram of the crotonyl-CoA biosynthesis pathway. (B) Scatter plot of *HIF-1α* versus *ACOX1* mRNA expression levels in OSCC samples from the TCGA cohort (*n* = 338, Pearson correlation). (C) qPCR analysis of *ACOX1* mRNA levels in OSCC cells under normoxia and hypoxia (24 h) (*P < 0.05 and **P < 0.01, unpaired *t* test). (D) Western blot analysis of the ACOX1 protein in OSCC cells under normoxia and hypoxia (24 h). β-Actin: loading control. (E) qPCR analysis of *ACOX1* mRNA levels in shRNA-transduced CAL27/HSC3 cells (**P < 0.01 and ***P < 0.001, one-way ANOVA). (F) Western blot analysis of HSP90AB1 K265cr in scramble control versus *ACOX1*-knockdown CAL27/HSC3 cells under normoxia/hypoxia (24 h). (G) Western blot analysis of HSP90AB1 K265cr in *ACOX1*-knockdown CAL27/HSC3 cells treated with crotonyl-CoA (50 μM) or butyryl-CoA (50 μM). (H) qPCR analysis of *ACOX1* mRNA levels in CAL27/HSC3 cells overexpressing ACOX1 (***P < 0.001, one-way ANOVA). (I) Western blot confirmation of ACOX1 protein levels in the overexpression system. (J) Western blot analysis of HSP90AB1 K265cr in CAL27/HSC3 cells overexpressing *ACOX1* under normoxia and hypoxia (24 h). (K) Diagram of the predicted HIF-1α-binding sites in the *ACOX1* promoter region (JASPAR database). (L) Dual-luciferase reporter activity of the ACOX1-WT promoter and mutant (ACOX1-MUT) under normoxia/hypoxia (**P < 0.01 and ***P < 0.001, one-way ANOVA). (M) Agarose gel electrophoresis of Cleavage Under Targets and Tagmentation (CUT&Tag) products using an anti-HIF-1α antibody. (N) CUT&Tag–qPCR analysis of the association of HIF-1α with the *ACOX1* promoter (***P < 0.001, unpaired *t* test). (O) Western blot analysis of ACOX1 and HSP90AB1 K265cr in CAL27/HSC3 cells treated with dimethyloxalylglycine (DMOG; 2 mM) or CAY10585 (CAY; 10 μM) under normoxia/hypoxia (24 h).

Having established the functional importance of HSP90AB1 K265cr in stabilizing TXN and counteracting hypoxic ROS, we next sought to elucidate the upstream regulatory mechanism responsible for inducing this modification in response to hypoxia. Given that lysine crotonylation requires crotonyl-CoA as the essential donor moiety [33] and that hypoxia profoundly reprograms cellular metabolism often through the master transcriptional regulator HIF-1α [15,17,27], we hypothesized that HIF-1α might orchestrate hypoxia-induced K265cr by modulating the availability of crotonyl-CoA or the expression of relevant modifying enzymes. By leveraging the TCGA database, we determined that among the key enzymes involved in crotonyl-CoA synthesis (Fig. 6A), *ACOX1* expression was positively correlated with *HIF-1α* levels (Fig. 6B and Fig. S6A to E).

To further investigate this relationship, we verified that hypoxic conditions promote ACOX1 expression at both the mRNA and protein levels in OSCC cell lines (Fig. 6C and D). Next, to assess the functional role of ACOX1 in hypoxia-induced crotonylation, we knocked down *ACOX1* in CAL27 and HSC3 cells via lentivirus-mediated shRNA (Fig. 6E and Fig. S6F). The results demonstrated that knockdown of *ACOX1* was sufficient to reverse the hypoxia-induced increase in HSP90AB1 K265cr in both the CAL27 and HSC3 cell lines, indicating a critical role of ACOX1 in this pathway (Fig. 6F). Interestingly, the addition of exogenous crotonyl-CoA, but not butyryl-CoA, restored the up-regulation of HSP90AB1 K265cr even in ACOX1-knockdown cells, suggesting that crotonyl-CoA is the direct substrate responsible for this modification (Fig. 6G). Furthermore, overexpression of *ACOX1* significantly increased HSP90AB1 K265cr levels even under normoxic conditions, confirming the role of ACOX1 as a key mediator in the modulation of crotonylation (Fig. 6H to J).

As a transcription factor, one of the key roles of HIF-1α lies in facilitating the transcription of its target genes. Using the JASPAR CORE (JASPAR) database to predict transcription-factor-binding sites, we identified potential HIF-1α-binding sites in the promoter region of *ACOX1* (Fig. 6K). To experimentally validate this interaction, we performed a dual-luciferase reporter assay in HEK293T cells. ACOX1 promoter constructs, including wild-type (ACOX1-WT) and mutant (ACOX1-MUT, with disrupted HIF-1α-binding sites), were generated. Following the transfection of the *HIF-1α* overexpression plasmid and hypoxic treatment, the luciferase activity driven by the ACOX1-WT promoter was significantly increased. In contrast, the HIF-1α-induced increase in luciferase activity was abolished in ACOX1-MUT, confirming that the effect was dependent on HIF-1α binding (Fig. 6L). To further verify this transcriptional regulation, Cleavage Under Targets and Tagmentation (CUT&Tag) assays were performed to assess HIF-1α binding at the promoter region of *ACOX1*. These results strongly suggest that HIF-1α specifically binds to the *ACOX1* promoter under hypoxia (Fig. 6M and N). Treatment of CAL27 and HSC3 cells with dimethyloxalylglycine (an HIF-1α stabilizer) under normoxic conditions resulted in notable up-regulation of both ACOX1 expression and HSP90AB1 K265cr. Conversely, the administration of methyl 3-[[2-[4-(2-adamantyl)phenoxy]acetyl]amino]-4-hydroxybenzoate (CAY10585, an HIF-1α degrader) abolished the hypoxia-induced increases in ACOX1 expression and HSP90AB1 K265cr, indicating that HIF-1α is indispensable for these effects. Moreover, under CAY10585 treatment, ACOX1 expression and HSP90AB1 K265cr levels under hypoxic conditions were comparable to those in the control group, further confirming the pivotal role of HIF-1α in this regulatory axis (Fig. 6O).

### Clinical and in vivo validation of the HIF-1α/ACOX1/HSP90AB1 K265cr/TXN axis

**Fig. 7.**
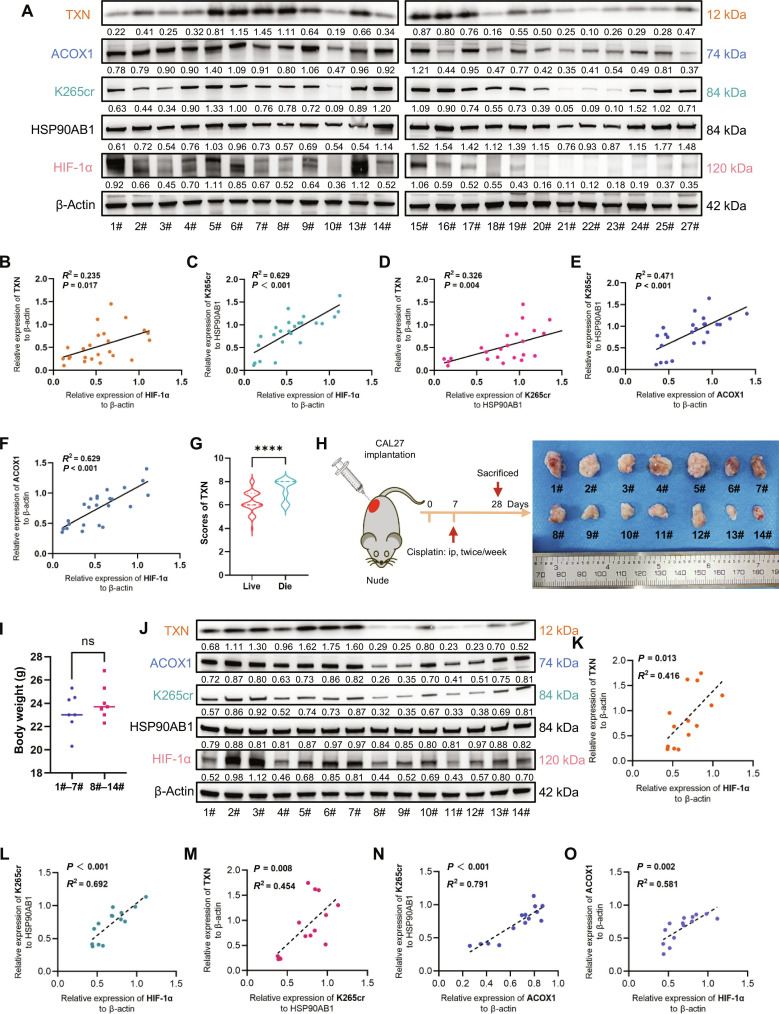
Clinical and in vivo validation of the HIF-1α/ACOX1/HSP90AB1 K265cr/TXN axis in OSCC. (A) Western blot analysis of the indicated proteins in 24 human OSCC tumor tissues. (B to F) Scatter plots of band intensity measurements from panel (A): (B) HIF-1α and TXN, (C) HIF-1α and HSP90AB1 K265cr, (D) HSP90AB1 K265cr and TXN, (E) ACOX1 and HSP90AB1 K265cr, and (F) HIF-1α and ACOX1 (*n* = 24, Pearson correlation). (G) TXN protein levels in 2 OSCC patient groups receiving postoperative chemoradiotherapy (*n* = 152; ****P < 0.0001, unpaired *t* test). (H) Schematic diagram of the cisplatin-treated xenograft experimental design (ip, intraperitoneal injection). (I) Body weight measurements of the mice during the treatment period (ns, not significant, unpaired *t* test). (J) Western blot analysis of the indicated proteins in xenograft tumor tissues. (K to O) Scatter plots of band intensity measurements from panel (J): (K) HIF-1α and TXN, (L) HIF-1α and HSP90AB1 K265cr, (M) HSP90AB1 K265cr and TXN, (N) ACOX1 and HSP90AB1 K265cr, and (O) HIF-1α and ACOX1 (*n* = 14, Pearson correlation).

Building upon the mechanistic delineation of the HIF-1α/ACOX1/HSP90AB1 K265cr/TXN signaling axis in OSCC, we next sought to validate its clinical relevance using patient-derived specimens. Protein extracts were prepared from a prospectively collected cohort of 27 OSCC tumor tissues. Following the quality assessment, 3 samples (nos. 11, 12, and 26) were excluded because of substantial protein degradation, resulting in 24 samples being available for subsequent analysis. Western blot analysis of these 24 clinical samples (Fig. 7A) revealed significant positive correlations between key axis components upon quantitative assessment of band grayscale intensities: HIF-1α expression correlated positively with both TXN (Fig. 7B) and HSP90AB1 K265cr levels (Fig. 7C). Crucially, the expression of HSP90AB1 K265cr was significantly positively associated with its downstream effector TXN (Fig. 7D) and its upstream regulator ACOX1 (Fig. 7E). Furthermore, ACOX1 expression itself correlated positively with HIF-1α (Fig. 7F). This interconnected pattern of correlations strongly supports the in vivo operation of the HIF-1α/ACOX1/HSP90AB1 K265cr/TXN signaling axis in human OSCC tumors. Given that postoperative chemoradiotherapy (particularly platinum-based regimens) for OSCC patients exerts tumoricidal effects primarily through ROS generation, our retrospective analysis of the institutional cohort (result 1) revealed significantly higher TXN protein expression in deceased patients than in survivors receiving postoperative chemoradiotherapy (Fig. 7G).

Complementary in vivo validation was performed using OSCC xenograft models treated with cisplatin (Fig. 7H and I and Fig. S7A). Western blot analysis of the harvested tumor tissues revealed the same positive correlation patterns among all axis components (Fig. 7J to O), effectively recapitulating the findings observed in the human clinical samples and reinforcing the biological importance of this signaling pathway.

### Targeting ACOX1 or TXN synergizes with cisplatin in OSCC

**Fig. 8.**
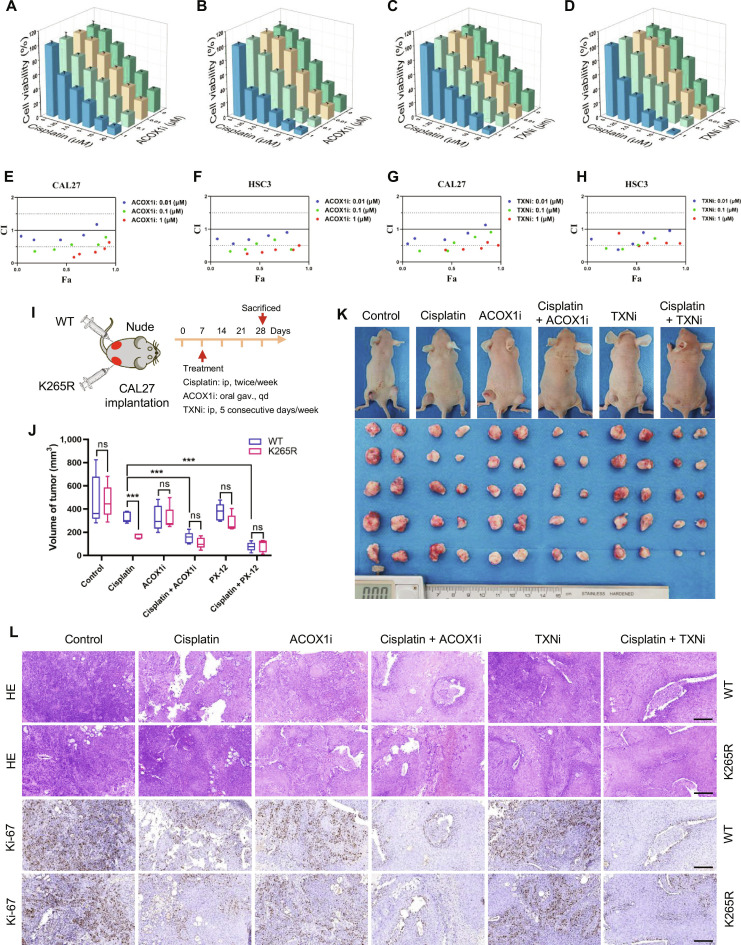
The dual targeting of ACOX1 or TXN synergizes with cisplatin to overcome therapeutic resistance in OSCC. (A and B) Cell viability measurements of (A) CAL27 and (B) HSC3 cells treated with combinations of cisplatin and 10,12-tricosadiynoic acid combinations. (C and D) Cell viability measurements of (C) CAL27 and (D) HSC3 cells treated with the combination of cisplatin and PX-12. (E to H) Combination index (CI) of the combination. The CI quantitatively depicts synergism (CI < 0.5), additive effects (0.5 < CI < 1), or antagonism (CI > 1) (Fa, fraction affected). (I) Schematic diagram of the in vivo drug combination experiment (ip, intraperitoneal injection; oral gav., oral gavage). (J) Tumor weight measurements across treatment groups (***P < 0.001; ns, not significant, paired *t* test and unpaired *t* test). (K) Representative photographs of mice and resected xenograft tumors. (L) Representative hematoxylin and eosin (HE) and Ki-67 IHC images of tumor sections (200×, scale bars, 100 μm).

Having established the functional importance of the HIF-1α/ACOX1/HSP90AB1 K265cr/TXN axis in redox regulation, we next sought to translate these mechanistic insights into a therapeutic strategy. Given that TXN is a well-established mediator of redox balance and drug resistance in various cancers [35,36] and that our data implicate ACOX1 as the upstream metabolic regulator enabling TXN stabilization via K265cr, we hypothesized that pharmacologically targeting either ACOX1 or TXN would synergize with cisplatin to overcome resistance in OSCC. We therefore evaluated the combined efficacy of cisplatin with specific inhibitors of ACOX1 (10,12-tricosadiynoic acid) and TXN (PX-12) both in vitro and in vivo. Synergy analysis demonstrated significant combinatorial inhibition of proliferation across most drug ratios (Fig. 8A to H), indicating robust cooperative effects.

For in vivo validation, nude mice received bilateral xenografts of CAL27 cells expressing either WT or K265R-mutant HSP90AB1 (Fig. 8I). Throughout the treatment period, body weights remained comparable across groups (Fig. S8A), confirming regimen tolerability. In the control group, the tumor volume and weight were comparable on both sides of the same mouse. In the cisplatin monotherapy group, tumors originating from WT cells were significantly larger in both volume and weight compared to those derived from K265R cells, which is consistent with the role of HSP90AB1 K265cr in mediating drug resistance. Strikingly, combination treatment with cisplatin and 10,12-tricosadiynoic acid or PX-12 significantly reduced the disparity in tumor growth between the WT and K265R cell-derived tumors (Fig. 8J and K and Fig. S8B and C).

To corroborate these observations, Ki-67 IHC revealed proliferation patterns mirroring tumor growth trends. Consistently, immunohistochemical staining for cleaved caspase-3, a marker of apoptosis, showed that the combination treatment enhanced tumor cell apoptosis compared to cisplatin alone. Compared with cisplatin alone, the combination treatment significantly reduced the difference in Ki-67 expression between WT and K265R tumors (Fig. 8L). Furthermore, the difference in cleaved-caspase-3-positive cells between the genotypes was also diminished in the combination treatment group, supporting the notion that the therapeutic efficacy involves the promotion of apoptosis (Fig. S8D). Collectively, these data establish dual targeting of ACOX1 or TXN with cisplatin as a promising strategy for overcoming cisplatin resistance in OSCC.

## Discussion

Our study elucidates a hypoxia-responsive metabolic–epigenetic axis that fundamentally redefines redox homeostasis in OSCC. The HIF-1α-driven transcriptional activation of ACOX1 establishes a critical metabolic bridge, elevating intracellular crotonyl-CoA to license site-specific crotonylation of HSP90AB1 at K265, a previously unrecognized regulatory switch. This mechanism aligns with the emerging recognition of hypoxia-induced PTMs as master regulators of cancer adaptation [18,37,38].

As an ATP-dependent molecular chaperone, HSP90AB1 mediates diverse cellular processes through client-protein interactions [23]. Our investigation specifically addressed why its K265cr modification exhibits apparent specificity for TXN within the broader hypoxia-induced crotonylome. This focus was guided by integrated bioinformatic analysis positioning TXN as a central node in the HSP90AB1 interactome, combined with clinical evidence of its posttranscriptional up-regulation, identified it as a compelling effector for mechanistic investigation. Functional validation confirmed that TXN overexpression rescues the redox and chemoresistance phenotypes caused by the K265R mutation. Given that HSP90 function is intricately regulated by PTMs [26] and that crotonylation modulates nonhistone protein activity [39–41], we hypothesized that K265cr enhances HSP90AB1–TXN interaction. We note that while our 100-ns simulation was sufficient to reveal these primary shifts, it represents a kinetic snapshot; longer simulations could further elucidate complex dynamics. Subsequent cellular assays confirmed that this modification strengthens the chaperone–client interaction, leading to TXN stabilization and functionally rescuing the redox and chemoresistance phenotypes caused by K265R mutation. Methodologically, we acknowledge the K265Q (Lys → Gln) mutagenesis used to mimic crotonylation via structural and charge similarity [42], offering experimental convenience; it cannot fully replicate the native modification’s reversible dynamics and distinct chemical properties. While our mechanistic investigation centered on TXN, it is plausible that K265cr serves as a broader regulatory switch, potentially modulating HSP90AB1’s chaperone activity toward other clients critical for hypoxic adaptation, a direct and promising avenue for future investigation.

Having established the functional impact of HSP90AB1 K265cr on TXN stabilization, we next delineate its upstream regulatory mechanism. We demonstrate that HIF-1α transcriptionally up-regulates ACOX1, a key enzyme in fatty acid β-oxidation, leading to an increased intracellular pool of crotonyl-CoA. However, the precise enzymology connecting ACOX1 activity to the site-specific crotonylation of HSP90AB1 at K265 remains unresolved. As ACOX1 itself is not a known crotonyltransferase, our data support a model wherein ACOX1-produced crotonyl-CoA “licenses” the modification, which is likely catalyzed by as-yet-unidentified crotonyltransferases. This gap in understanding the complete enzymatic circuitry represents an important boundary of our current model and a specific direction for future research. Notably, this model of a metabolic enzyme shaping the crotonyl-CoA pool to regulate specific protein crotonylation finds a parallel in breast cancer, where enoyl-CoA hydratase 1 (ECHS1)-mediated degradation of crotonyl-CoA reduces the crotonylation of phosphoglycerate kinase 1 (PGK1), thereby promoting glycolysis [43].

Functionally, the HIF-1α/ACOX1/HSP90AB1 K265cr/TXN axis acts as a precision rheostat for ROS calibration in OSCC. By stabilizing TXN, it maintains ROS within a narrow protumorigenic window that sustains proliferation while averting cytotoxicity. This redox fine-tuning confers a robust survival advantage, directly exemplified by the marked cisplatin resistance observed in tumors with an intact axis, a phenotype reversible through genetic (K265R) or pharmacological disruption. The translational promise of this work lies in its direct confrontation of therapeutic resistance. We demonstrate potent synergy when combining cisplatin with an ACOX1 inhibitor [44] or a TXN inhibitor [36], effectively neutralizing the redox-adaptive advantage. This strategy is consistent with approaches that target redox-adaptive pathways to overcome treatment resistance [45]. Importantly, while focused on cisplatin, this axis may confer cross-resistance to other ROS-inducing therapies such as radiotherapy. Beyond these cell-autonomous effects, this modestly elevated yet controlled ROS state likely shapes the immunosuppressive TME characteristic of OSCC, a prototypical “immune-cold” malignancy [46]. A stabilized ROS level may fail to provide the dynamic oxidative signals required for optimal antigen-presenting cell activation and T-cell priming while also potentially favoring the recruitment of immunosuppressive cells like M2-like tumor-associated macrophages [47,48]. Thus, targeting this axis presents a dual therapeutic opportunity: disrupting redox-driven chemoresistance while potentially reversing local immune suppression. Future work should directly investigate whether its inhibition can remodel the TME and synergize with immunotherapies to “ignite” anti-tumor immunity in OSCC.

We must acknowledge several limitations inherent to our study. First, as a specialized surgical center, our institution does not administer first-line platinum-based chemotherapy for OSCC. Consequently, while we correlate axis activation with aggressive disease, we lack the clinical data to directly correlate ACOX1 expression or HSP90AB1 K265cr levels with cisplatin response or recurrence after platinum-based therapy within our cohort. This is a key translational gap we plan to address in future collaborative studies with comprehensive chemotherapeutic records. Second, exploring innovative cisplatin delivery strategies (e.g., nanoparticle formulations and local drug-eluting systems) represents a promising interdisciplinary avenue to enhance therapeutic efficacy and reduce toxicity, which was beyond the scope of this mechanistic study but is a crucial consideration for clinical translation.

**Fig. 9.**
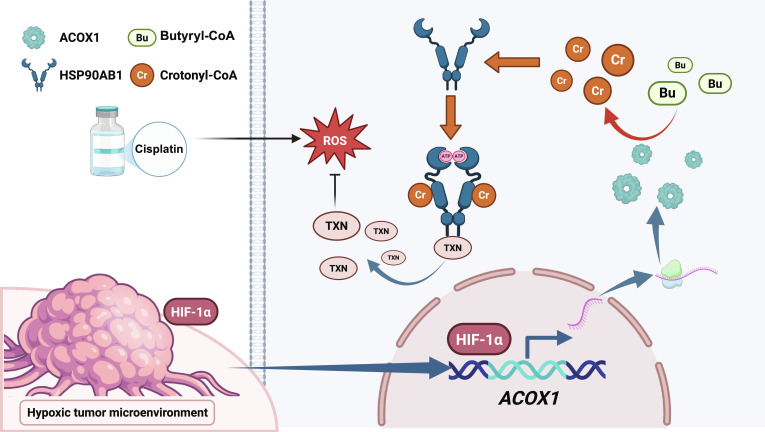
The schematic diagram of the HIF-1α/ACOX1/HSP90AB1 K265cr/TXN signaling axis in OSCC.

In summary, this study delineates a hypoxia-driven HIF-1α/ACOX1/HSP90AB1 K265cr/TXN signaling axis that maintains protumorigenic ROS homeostasis in OSCC (Fig. 9). We provide a mechanistic framework linking metabolic reprogramming, a specific protein crotonylation event, chaperone function, and redox effector stabilization. Our work not only reveals a fundamental adaptive mechanism in therapy-resistant OSCC but also provides a preclinical rationale for cotargeting this axis to overcome redox-mediated chemoresistance. Future work will explore the enzymology of the modification, its role in regulating additional client networks, its impact on the tumor immune microenvironment, and its relevance across other ROS-inducing cancer therapies.

## Materials and Methods

### Cell lines

The OSCC cell lines CAL27, HSC3, and HN6 were purchased from Shanghai Zhongqiao XINZHOU Biotechnology Co., Ltd. The nontumorigenic oral epithelial cell line DOK was obtained from the Nanjing University Institute of Stomatology. Prior to experimentation, all cell lines were authenticated using short tandem repeat profiling (Genetic Testing Biotechnology, Suzhou, China) and screened for mycoplasma contamination with the Myco-Search One-Step Mycoplasma Detection Kit (Vazyme, Nanjing, China) to ensure the absence of contamination. OSCC cells were maintained in Dulbecco’s modified Eagle’s medium (DMEM) (KeyGEN, Nanjing, China), while DOK cells were cultured in Roswell Park Memorial Institute 1640 medium (KeyGEN, Nanjing, China). Both media were supplemented with 10% fetal bovine serum (BioInd, Israel) and 1% penicillin–streptomycin (KeyGEN, Nanjing, China). Cultures were maintained in a humidified incubator at 37 °C under either normoxic conditions (5% CO_2_ and 20% O_2_) or hypoxic conditions (5% CO_2_, 1% O_2_, and 94% N_2_). Once the cells reached confluency, they were subcultured with 0.05% trypsin containing 0.02% EDTA (KeyGEN, Nanjing, China).

### Reagents

The following reagents were used in this study: 10,12-tricosadiynoic acid (MedChemExpress, HY-135425; CAS 66990-30-5), PX-12 (Selleck, S7947; CAS 141400-58-0), cisplatin (Selleck, S1166; CAS 15663-27-1), NVP-BEP800 (Selleck, S1498; CAS 847559-80-2), dimethyloxalylglycine (Selleck, S7483; CAS 89464-63-1), CAY10585 (Selleck, S8441; CAS 934593-90-5), CHX (Selleck, S7418; CAS 66-81-9), crotonic acid (Sigma-Aldrich, 113018; CAS 107-93-7), crotonyl-CoA (Sigma-Aldrich, 28007; CAS 102680-35-3), and butyryl-CoA (Sigma-Aldrich, B1508; CAS 102282-28-0).

### Human OSCC tissues

The use of human samples in this study was approved by the Research Ethics Committee of Nanjing Stomatology Hospital (Approval Number: NJSH-2023NL-093-1). All procedures involving human samples were conducted in strict accordance with the ethical guidelines outlined in the Declaration of Helsinki.

### Measurement of ROS

The intracellular ROS levels were quantified via the Reactive Oxygen Species Assay Kit (Beyotime Biotechnology, Shanghai, China). Following experimental treatments, the cells were incubated with 10 μM DCFH-DA at 37 °C for 20 min. After 2 washes with phosphate-buffered saline (PBS), ROS detection was performed through 2 complementary approaches. For qualitative imaging, the cells were visualized via a confocal laser scanning microscope (Nikon Ti; Nikon Instruments Inc., Melville, NY, USA). For quantitative analysis, the cell suspensions were prepared for counting, and the fluorescence intensity was measured via a microplate reader (SpectraMax M3; Molecular Devices, San Jose, CA, USA) at identical wavelengths.

### RNA extraction and reverse transcription qPCR

Total RNA was extracted via the Super FastPure Cell RNA Isolation Kit (Vazyme, Nanjing, China) according to the manufacturer’s protocol. Reverse transcription was then performed via HiScript IV RT SuperMix with gDNA Eraser (Vazyme, Nanjing, China) to synthesize complementary DNA. Quantitative real-time polymerase chain reaction (qPCR) was carried out on an Applied Biosystems ViiA7 instrument with the ChamQ Universal SYBR qPCR Master Mix (Vazyme, Nanjing, China) following the manufacturer’s instructions. Gene expression levels were calculated via the 2^−ΔΔCt^ method. The sequences of the primers used in this study are provided in Table S1.

### Protein extraction and Western blotting

Following the indicated treatments, the cells and tumor tissues were lysed in ice-cold radioimmunoprecipitation assay (RIPA) buffer (Beyotime Biotechnology, Shanghai, China) supplemented with a protease inhibitor cocktail (Novizan Biotechnology, Suzhou, China). For tissue samples, homogenization was performed with RIPA buffer at 10 μl/mg tissue prior to lysis. The lysates were centrifuged at 12,000×g for 15 min at 4 °C, and the supernatants were collected for protein quantification via a bicinchoninic acid assay kit (KeyGEN BioTECH, Nanjing, China). Next, the protein samples were mixed with 5× loading buffer (Beyotime, Shanghai, China) and heated at 95 °C for 10 min, after which each product was separated via sodium dodecyl sulfate–polyacrylamide gel electrophoresis (Smart Life Sciences, Changzhou, China). The proteins were subsequently transferred to 0.45-mm polyvinylidene difluoride membranes (Millipore, USA) and blocked with 5% fat-free milk in Tris-buffered saline with Tween-20 (TBST) at room temperature for 2 h. The membranes were subsequently incubated with primary antibodies overnight at 4 °C and then with horseradish peroxide (HRP)-conjugated secondary antibodies at room temperature for 2 h, followed by exposure to an enhanced chemiluminescence reagent (Vazyme, Nanjing, China). Images were captured via Tanon 6200 Luminescent Imaging Workstation (Tanon, Shanghai, China). The following primary antibodies were used: rabbit anti-β-actin (1:4,000; Proteintech, 20536-1-AP), rabbit anti-TXN (1:1,500; Abways, CY6679), rabbit anti-HIF-1α (1:1,500; Abways, CY5197), rabbit anti-HSP90AB1 (1:10,000; Abcam, ab203085), rabbit anti-pan-crotonyllysine (1:1,000; PTM Biolab, PTM-501), rabbit anti-Flag (1:5,000; Abways, AB0030), mouse anti-His (1:10,000; Proteintech, 66005-1-Ig), rabbit anti-HSP90AB1 K265cr (1:1,000; PTM Biolab), and rabbit anti-ACOX1 (1:4,000; Proteintech, 10957-1-AP). The secondary antibody used was HRP-conjugated goat anti-rabbit IgG (H+L) (1:8,000; Proteintech, SA00001-2).

### Immunohistochemistry

IHC was performed as described previously with the following modifications [49]: The tissue sections were incubated overnight at 4 °C with the following primary antibodies: rabbit anti-TXN (1:400; Abways, CY6679), rabbit anti-HIF-1α (1:200; Abways, CY5197), rabbit anti-Ki-67 (1:2,000; Proteintech, 84192-4-RR), and mouse anti-cleaved caspase 3 (1:2,000; Proteintech, 68773-1-Ig). The IHC staining scores of TXN and HIF-1α were evaluated via the Allred semiquantitative scoring system. The proportion of positive tumor cells (proportion score) was graded as 0 (none), 1 (<1%), 2 (1% to 10%), 3 (11% to 33%), 4 (34% to 66%), or 5 (>66%). The staining intensity (intensity score) was scored as 0 (negative), 1 (weak), 2 (moderate), or 3 (strong). A composite Allred score (range 0 to 8) was calculated by summing the proportion scores and intensity scores. All slides were independently assessed by 2 blinded pathologists, with discrepancies resolved through consensus review via a multiheaded microscope.

### Multiplexed IHC

Multiplex IHC was performed via the TSA Fluorescence Triple Staining Plus Kit (RK05903P, ABclonal, China). Briefly, OSCC tissue sections were deparaffinized, rehydrated, and washed in TBST buffer. Antigen retrieval was conducted in preheated citrate buffer (100 °C, 20 min). After blocking endogenous peroxidase activity and nonspecific binding sites, the sections were sequentially stained with an anti-TXN antibody (1:400; Abways, CY6679)/TSA 520, an anti-8-OHdG antibody (1:200; Bioss, bs-1278R)/TSA 570, and an anti-HIF-1α antibody (1:200; Abways, CY5197)/TSA 690. The tissue sections were incubated overnight at 4 °C with the primary antibody. For each cycle, the tissue sections were incubated with HRP-conjugated anti-rabbit/mouse secondary antibodies at room temperature for 50 min, followed by incubation with the corresponding tyramine substrates for 4 min. Between cycles, the antibodies were stripped with antibody elution buffer (RM02984P, ABclonal, China) before proceeding to the next cycle. The sections were subsequently counterstained with 2 drops of 4′,6-diamidino-2-phenylindole (DAPI), rinsed with distilled water, and manually coverslipped. After air-drying, the sections were analyzed via laser scanning confocal microscopy (Nikon Ti; Nikon Instruments Inc., Melville, NY, USA).

### TCGA data acquisition and processing

Transcriptomic data and corresponding clinical annotations for head and neck squamous cell carcinoma were obtained from the TCGA database via the Genomic Data Commons Data Portal (https://portal.gdc.cancer.gov/). The cases were subsequently filtered to include only OSCC samples for analysis. TXN expression levels were derived from log_2_-transformed fragments per kilobase of transcript per million mapped reads (FPKM) values and categorized into high/low groups using the median expression level as the cutoff. Associations between TXN expression and clinicopathological parameters (T stage, N stage, pathological stage, and grade) were evaluated via the Kruskal–Wallis test and Wilcoxon rank-sum test. Kaplan–Meier analysis with log-rank tests was employed to assess survival differences. Concurrently, Pearson correlation coefficients were calculated to examine the relationships between HIF-1α and ACOX1/ACOX3/GCDH/ACSS2/ACADS/ECHS1 expression across the OSCC samples, with the resulting *P* values adjusted for multiple testing via the Benjamini–Hochberg method. All analyses were performed in R (v4.2.2) via the TCGAbiolinks package for data retrieval and the survival and ggplot2 packages for statistical analysis and visualization.

### Co-IP and MS analysis

The co-IP assay was conducted via the Pierce Crosslink Magnetic IP/Co-IP Kit (Thermo Fisher Scientific Inc., 88805) according to the manufacturer’s protocol. The cell lysates were subsequently centrifuged at 13,000×g for 15 min at 4 °C to remove intact cells and debris. The supernatant was then incubated overnight at 4 °C with rabbit anti-HSP90AB1 (1:100; Abcam, ab203085), rabbit anti-TXN (1:50; Abways, CY6679), or rabbit IgG isotype control recombinant antibody (Proteintech, 98136-1-RR). Magnetic beads cross-linked to antibodies were prepared in advance and used to capture the immune complexes. After incubation, the beads were collected using a magnetic stand, and unbound proteins were removed through a series of washes. Finally, the immune complexes were eluted with elution buffer for subsequent analysis. MS identification was carried out by GeneCreate Biological Engineering Co., Ltd. (Wuhan, China).

### Plasmids and transfection

The human *HSP90AB1* expression plasmids, including the Flag-tagged WT and the mutant K265 (K265R: lysine [K] to arginine [R]; K265Q: lysine [K] to glutamine [Q]), along with the human *ACOX1* and *TXN* expression plasmids, were synthesized and encapsulated into lentiviruses by Zebrafish Biotech Co., Ltd. (Nanjing, China). Silent mutations were engineered into the rescued *HSP90AB1* sequence (5′-CGCATGGAAGAAGTCGATTAG-3′) to evade shRNA targeting without altering the protein sequence. Transfection experiments were conducted according to the manufacturer’s protocol.

### Lentiviruses and transfection

The knockdown of *HSP90AB1* or *ACOX1* in CAL27 and HSC3 cells was achieved via a lentiviral vector containing a shRNA specifically targeting *HSP90AB1* or *ACOX1* following the manufacturer’s protocol (Zebrafish Biotech, Nanjing, China). The sequences of the shRNAs used in this study were as follows: sh*HSP90AB1*, 5′-CGCATGGAAGAAGTCGATTAG-3′; sh*ACOX1* #1, 5′-CGAAAGCCTAACCGAAGCATA-3′; sh*ACOX1* #2, 5′-CGCTGAGTAACAAGCTGACTT-3′; and sh*ACOX1* #3, 5′-GCCTGGAACTTGGAGATCATT-3′. Details regarding the transfection procedures were described in our previous reports [50].

### CCK-8 assays

For the cell viability assay, a total of 3,000 cells in a volume of 100 μl per well were seeded into a 96-well plate, with 6 replicate wells per condition. After allowing the cells to adhere overnight, the culture medium was replaced with media containing the appropriate reagents on the basis of the treatment conditions. The cells were then cultured for an additional 48 h. To assess cell viability, a working solution was prepared by mixing 10 μl of CCK-8 reagent (New Cell & Molecular Biotech, Suzhou, China) with 100 μl of DMEM. A total of 110 μl of this mixture was added to each well and incubated for 1 to 1.5 h. The optical density (OD) at 450 nm was measured via a SpectraMAX M3 microplate reader (Molecular Devices, San Jose, CA, USA).

### EdU incorporation assays

The EdU incorporation assay was conducted according to the manufacturer’s instructions (KeyGEN, Nanjing, China) to evaluate cell proliferation. Briefly, cells were seeded into 28.2-mm glass-bottom culture dishes and cultured for 24 h with the specified treatments. The cells were subsequently incubated with 50 μM EdU for 2 h at 37 °C. Following EdU incorporation, the cells were fixed with 4% formaldehyde for 30 min and permeabilized with 0.5% Triton X-100 for 10 min at room temperature. The cells were subsequently treated with a 1× Apollo reaction cocktail for 30 min. Finally, the nuclei were stained with Hoechst 33342, and the fluorescence signals were visualized via a laser scanning confocal microscope (Nikon Ti; Nikon Instruments Inc., Melville, NY, USA).

### Colony formation assays

The resuspended cells were seeded into 6-well plates at a density of 2,000 cells per well for CAL27 cells and 800 cells per well for HSC3 and HN6 cells. The cells were allowed to adhere overnight and then cultured to form colonies for 10 d under the specified treatment conditions. Next, the cells were fixed and stained with crystal violet (Beyotime, Shanghai, China). Images of the stained colonies were captured via a scanner (EPSON V330, Beaverton, OR, USA). The area of the colonies was quantified via the ImageJ software.

### His pull-down assays

The His-tagged HSP90AB1 fusion protein and His peptide were purchased from Cusabio Technology Co., Ltd. (Wuhan, China) and Yeasen Biotechnology Co., Ltd. (Shanghai, China). The in vitro pull-down assay was performed via a commercial kit (FitGene Technology Co., Ltd., Catalog No. FI8805) following the manufacturer’s instructions. Briefly, TXN-overexpressing HEK293T cells (2 × 10^7^ cells) were collected and disrupted via sonication, and the lysates were kept on ice. Next, 50 μg of His peptide (control group) or His-HSP90AB1 (experimental group) was incubated with 50 μl of His-tagged purification resin at 4 °C for 2 h. The cell lysates were divided equally into 2 parts and mixed with either the control or experimental resin, followed by overnight incubation on a rotating mixer at 4 °C. Finally, the bound proteins were eluted and collected for Western blot analysis.

### Immunofluorescence assays

The cells that adhered to the surface of the glass-bottom culture dishes were fixed with cold methanol at −20 °C for 10 min. Subsequently, the cells were blocked with 3% bovine serum albumin for 30 min. Next, the cells were incubated with the appropriate primary antibodies at 4 °C overnight, followed by a 1-h incubation with the secondary antibody at room temperature. Finally, the DNA was stained with DAPI. Images were taken via a confocal laser scanning microscope (Nikon Ti). The following primary antibodies were used: mouse anti-HSP90AB1 (1:250; Invitrogen, 37-9400) and rabbit anti-TXN (1:100; Invitrogen, MA5-29625). The secondary antibodies used in this study were goat anti-mouse IgG (H+L) Superclonal secondary antibody, Alexa Fluor 488 (1:2,000; Invitrogen, A28175SAMPLE), and goat anti-rabbit IgG (H+L) cross-adsorbed ReadyProbes secondary antibody, Alexa Fluor 594 (2 drops per mL; Invitrogen, R37117).

### Molecular docking and molecular dynamics simulation

Molecular docking and molecular dynamics simulations were conducted with technical support from the Scientific Compass Testing Platform. The crystal structures of HSP90AB1 and TXN were preprocessed via PyMOL by removing heteroatoms and repairing incomplete regions. Subsequently, protein–protein docking between HSP90AB1 and TXN was performed via the HDOCK software. The conformation with the highest docking score was selected for further analysis. Molecular dynamics simulations were carried out via Gromacs 2021 with the amber99sb force field. The system was constructed by placing the protein complex in a simulation box, which was filled with water molecules via the TIP3P water model. Sodium (Na^+^) and chloride (Cl^−^) ions were added to neutralize the overall charge of the system. The simulations were conducted under physiological conditions, with the temperature set to 298.15 K and the pressure maintained at 1 bar. Prior to the production phase, energy minimization was performed to resolve steric clashes, followed by 100 ps of NVT equilibration and 100 ps of NPT equilibration to ensure system stability. A 100-ns production simulation was then carried out via the leapfrog algorithm for integrating Newton’s equations of motion with a time step of 2 fs. The temperature coupling was controlled via the V-rescale thermostat, whereas the Parrinello–Rahman barostat was used for pressure coupling. Neighbor searching was performed via the Verlet algorithm, with cutoff radii for Coulombic and van der Waals interactions set to 1.4 nm. Long-range electrostatic interactions were calculated via the particle–mesh Ewald method, and long-range dispersion corrections were applied to the energy and pressure. This workflow enabled a detailed investigation of the interactions and dynamic behaviors of the HSP90AB1–TXN complex, providing insights into its stability and functional properties under physiological conditions.

### Dual-luciferase reporter gene assays

The binding sequences between the ACOX1 gene promoter and HIF-1α were predicted via the JASPAR online database (http://jaspar.genereg.net/). Firefly luciferase plasmids containing either the WT *ACOX1* promoter sequence or site-mutant sequences were synthesized by Zebrafish Biotech Co., Ltd. (Nanjing, China). Additionally, the *Renilla* luciferase plasmid and *HIF-1α* expression plasmid were provided by the same company. For the transfection experiments, HEK293T cells were grown to 50% to 70% confluence, and the plasmids were transfected with Lipofectamine 8000 reagent (Beyotime, Shanghai, China) following the manufacturer’s protocol. After 48 h of transfection, dual-luciferase activity was measured via the Dual Luciferase Reporter Gene Assay Kit (Yeasen Biotechnology, Shanghai, China) in compliance with the kit instructions.

### CUT&Tag assays

The CUT&Tag assay was carried out via the Hieff NGS In Situ DNA Binding Profiling Library Prep Kit for Illumina V2 (Yeasen Biotechnology, Shanghai, China) according to the manufacturer’s instructions. In brief, 50,000 verified CAL27 or HSC3 cells were immobilized directly onto activated Con A magnetic beads and permeabilized with digitonin. The cells were subsequently incubated with primary and secondary antibodies at a 1:100 dilution. To facilitate the binding of the pA/G-Tn5 transposase, the samples were further incubated with a 1:50 dilution of the pA/G-Tn5 adapter complex and activated to fragment chromatin in situ. DNA fragments were then extracted via DNA extraction beads, enriched via PCR, and subsequently used for library generation. Amplified and purified DNA fragments were subsequently analyzed through qPCR and additional PCR assays. The primers used in this study to target the *ACOX1* promoter were as follows: *ACOX1*-F, GGAGGGGCGGGATACAAAAA, and *ACOX1*-R, TCTTTCTCCGTGGCCCTTTG.

### Animal experiments

The animal experiments described in this study were conducted in full compliance with the guidelines set by the Animal Care and Use Committee of the Sciences and Technology Ethics Committee of Nanjing University (approval number: IACUC-D2401014). All protocols adhered strictly to the ethical principles outlined in the National Institutes of Health Guide for the Care and Use of Laboratory Animals to ensure the humane treatment of the animals used in the research.

In the first experimental setup, a subcutaneous tumor model was established by injecting 1 × 10^7^ CAL27 cells suspended in 100 μl of PBS into the right flanks of 5-week-old male BALB/c nude mice (Charles River Laboratories, Beijing, China). Seven days postinoculation, the mice were treated with 2 mg/kg cisplatin administered intraperitoneally twice per week for a total duration of 3 weeks. At the end of the treatment period, the mice were sacrificed, and the xenograft tumors were excised. The tumor volumes and weights were accurately measured. Each tumor was then bisected, with one-half being fixed and embedded for pathological analysis and the other half being cryopreserved at −80 °C for further biochemical studies.

For the drug combination therapy experiment, 1 × 10^7^ CAL27 cells transfected with the HSP90AB1 WT or K265R-mutant plasmids were injected into both flanks of 5-week-old male BALB/c nude mice (left flank: WT; right flank: K265R) in 100 μl of a PBS suspension. After 7 d, the mice were randomly assigned to 6 groups (*n* = 5 per group) and subjected to the appropriate treatments. Drug administration was performed as follows: cisplatin (2 mg/kg, intraperitoneal, twice weekly), 10,12-tricosadiynoic acid (200 μg/kg, oral gavage, daily), and PX-12 (12 mg/kg, intraperitoneal, 5 consecutive days/week). Control groups received corresponding vehicles with identical administration schedules and routes. Treatments were administered for a duration of 21 d, after which the mice were euthanized, followed by excision of the xenograft tumors. The tumor volumes and weights were recorded, and the tumors were again bisected for further analysis. One-half of each tumor sample was fixed and embedded for pathological examination, while the other half was stored at −80 °C for subsequent molecular or biochemical analyses.

### Statistical analysis

All data are presented as mean ± standard deviation. Statistical comparisons were conducted via a 2-sided Student *t* test for pairwise analysis or one-way analysis of variance for multiple group comparisons when applicable. Statistical analyses were performed via the SPSS 26.0 software package (SPSS Inc., IL, USA), with a significance threshold set at *P* < 0.05. Graphs and data visualizations were generated via GraphPad Prism 9 (GraphPad Software Inc., San Diego, CA, USA).

## Data Availability

All reagents used in this work are available upon request and a brief statement describing the purpose for their use. The data that support the findings of this study are available in the Supplementary Materials of this article.
