# Supplementary material for: Hypoxic Reprogramming of ACOX1-Driven HSP90AB1 Crotonylation Stabilizes Thioredoxin to Orchestrate Redox Homeostasis in Oral Squamous Cell Carcinoma
Source: Research (Wash D C). 2026 Feb 10;9:1129. doi: 10.34133/research.1129 (PMC12886717; doi:10.34133/research.1129)
Supplement: Supplementary 1 — Figs. S1 to S8 Table S1 [file research.1129.f1.docx]

Supplementary Materials for

**Hypoxic Reprogramming of ACOX1-Driven HSP90AB1 Crotonylation Stabilizes Thioredoxin to Orchestrate Redox Homeostasis in Oral Squamous Cell Carcinoma**

**This file includes:**

Supplementary Figures. S1 to S8

Supplementary Table. S1


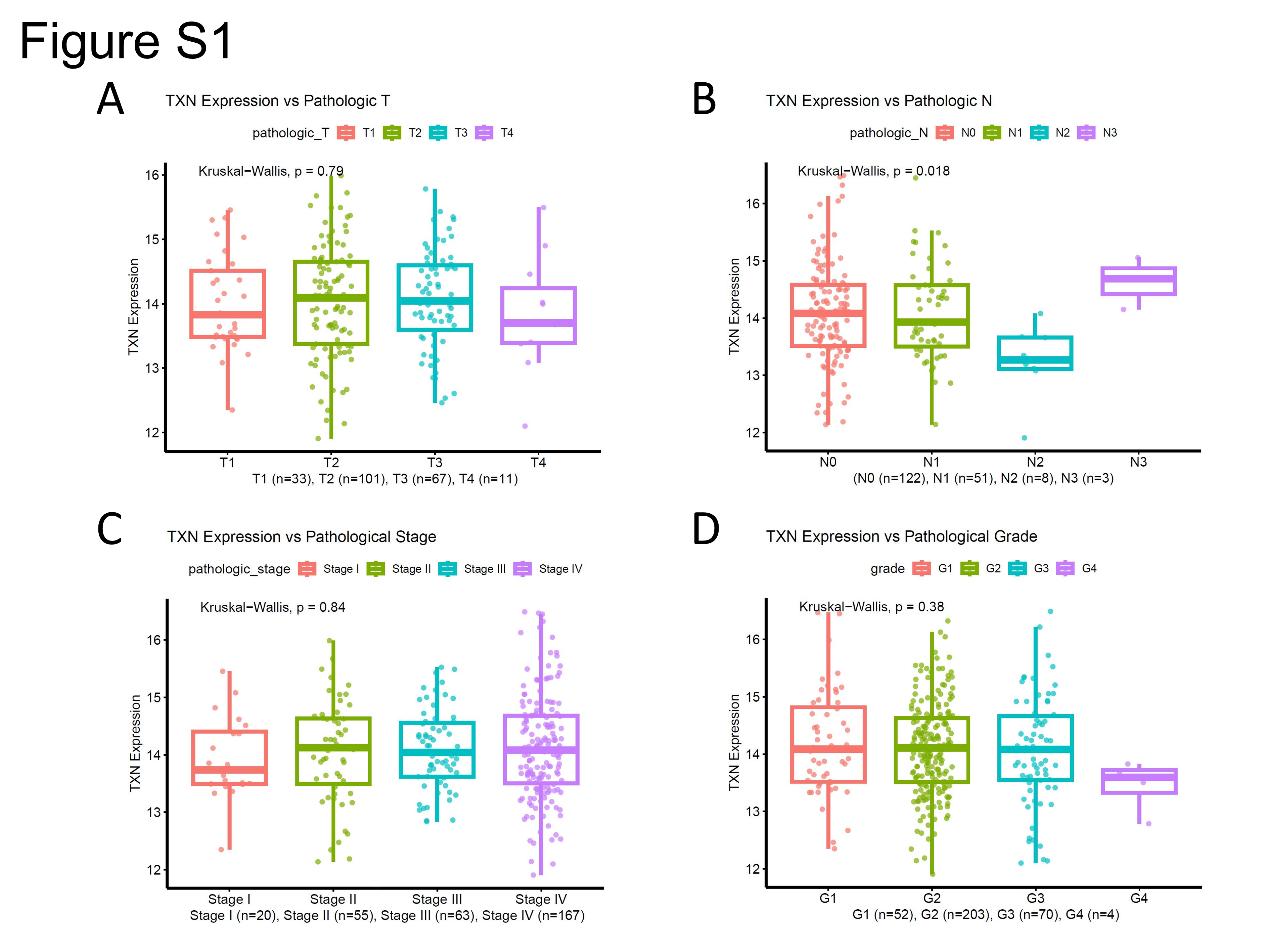


**Supplementary Figure 1: Association between *TXN* mRNA expression and clinicopathological staging in TCGA-OSCC cohort**

(A-D) *TXN* mRNA expression levels in OSCC tumors grouped by (A) T stage, (B) N stage, (C) pathological stage, (D) histological grade from the TCGA-OSCC cohort.


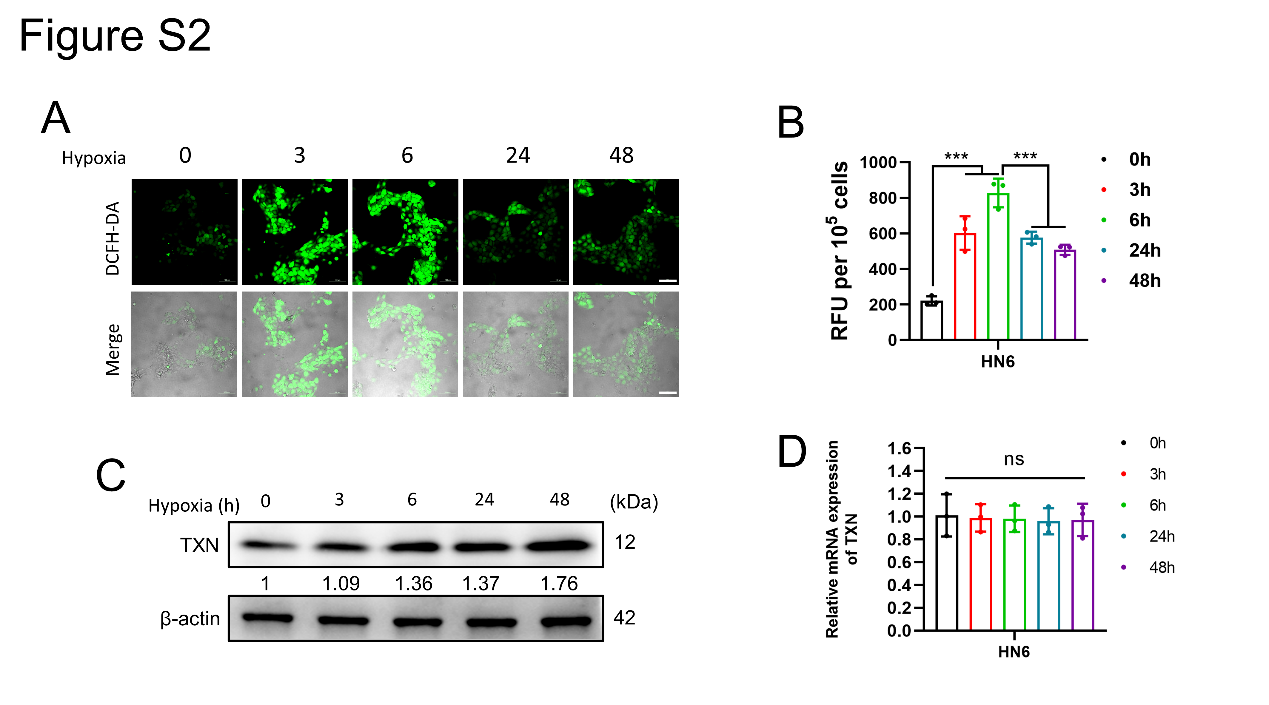


**Supplementary Figure 2: Dynamic changes in ROS levels and TXN expression in HN6 cells under hypoxic conditions**

(A) Representative confocal microscopy images of DCFH-DA staining in HN6 cells under hypoxic conditions at indicated time points (hours) (200X,scale bars, 100 µm).

(B) Quantification of DCFH-DA fluorescence intensity in HN6 cells during hypoxic exposure (***P<0.001, one-way ANOVA).

(C) Western blot analysis of TXN protein expression in HN6 cells under hypoxia at indicated durations (hour). β-actin serves as loading control.

(D) qPCR analysis of *TXN* mRNA levels in HN6 cells during hypoxic exposure (ns: not significant, one-way ANOVA).


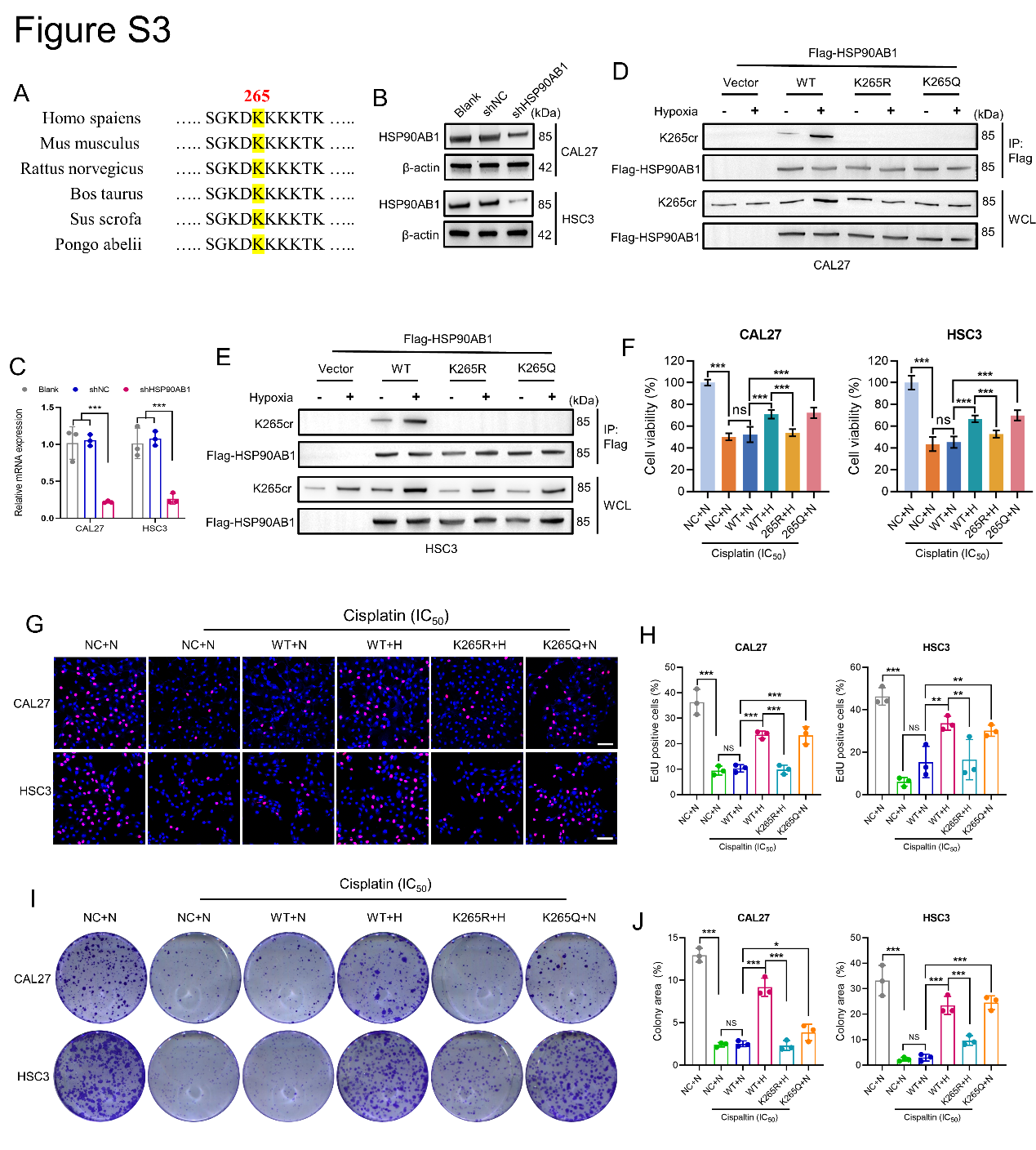


**Supplementary Figure 3: Site-specific K265cr modification of HSP90AB1 drives hypoxia-induced cisplatin resistance in OSCC**

(A) Sequence alignment of the HSP90AB1 K265 region across multiple species.

(B) Western blot analysis of HSP90AB1 protein in shRNA-transduced CAL27/HSC3 cells. β-actin serves as loading control.

(C) qPCR analysis of *HSP90AB1* mRNA levels in shRNA-transduced CAL27 and HSC3 cells (***P<0.001, one-way ANOVA).

(D-E) IP of Flag-HSP90AB1 in HSP90AB1-knockdown (D) CAL27 and (E) HSC3 cells reconstituted with HSP90AB1 variants under normoxia/hypoxia (24h), probed with site-specific K265cr antibodies.

(F) Cell viability measurements in cells expressing HSP90AB1 variants treated with cisplatin under normoxia and hypoxia (24h) (ns: not significant, ***P<0.001, one-way ANOVA).

(G) Representative EdU incorporation images (red: EdU signal; blue: DAPI) in indicated conditions (400X,scale bars, 50 µm).

(H) Quantification of EdU-positive cells from panel G experiments (ns: not significant, **P<0.01, ***P<0.001, one-way ANOVA).

(I) Representative colony formation images in indicated conditions.

(J) Quantified colony area measurements from panel I experiments (ns: not significant, *P<0.1, ***P<0.001, one-way ANOVA).


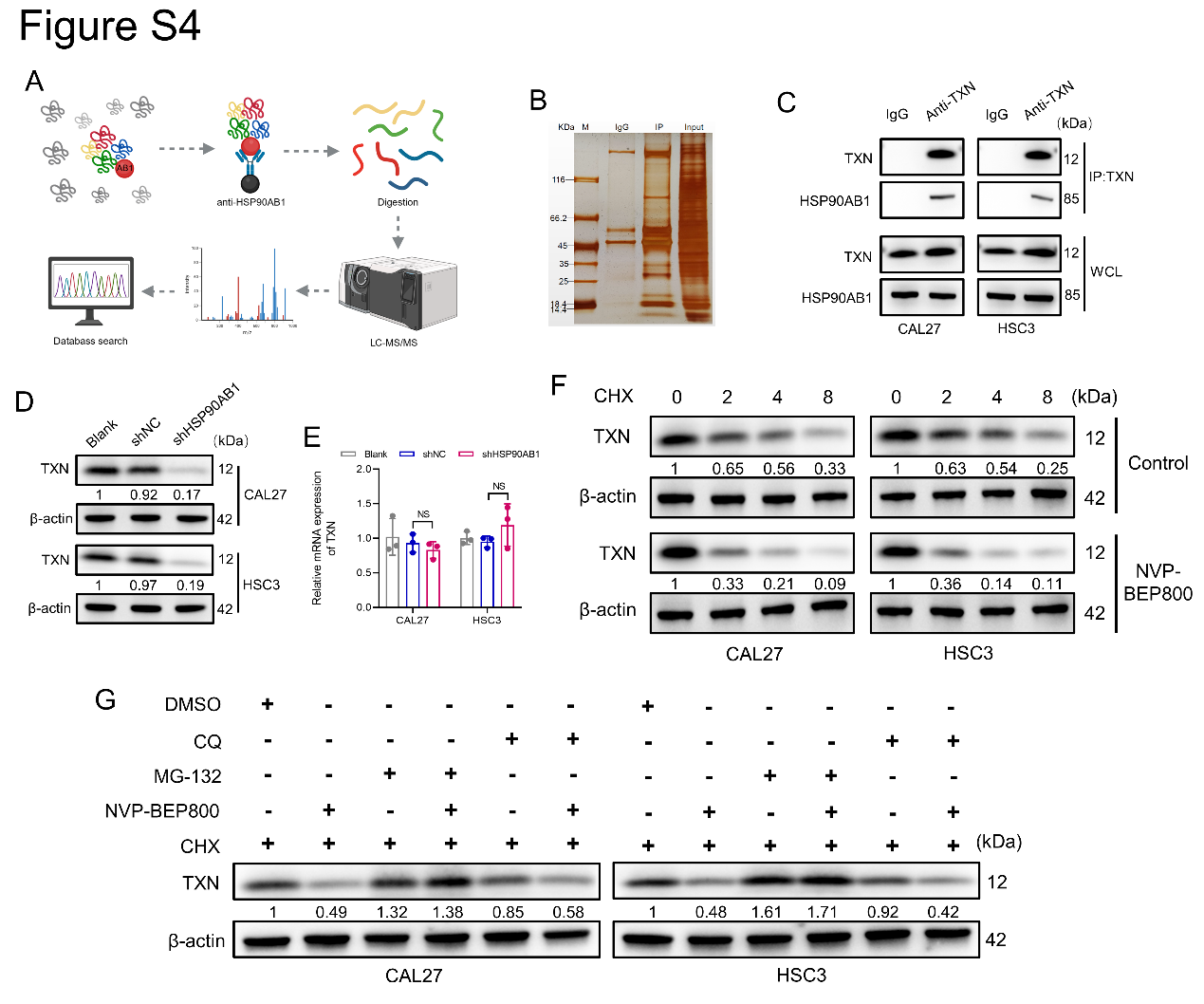


**Supplementary Figure 4: HSP90AB1 physically interacts with and stabilizes TXN protein.**

(A) Workflow diagram of co-IP coupled with mass spectrometry (Co-IP-MS) for HSP90AB1 interactor identification.

(B) Silver staining of immunoprecipitated complexes from CAL27 cells using anti-HSP90AB1 antibody.

(C) Co-IP of endogenous HSP90AB1 and TXN in CAL27 and HSC3 cells. IgG: control antibody; WCL: whole-cell lysate.

(D) Western blot analysis of TXN protein levels in shHSP90AB1 versus shCtrl CAL27/HSC3 cells. β-actin: loading control.

(E) qPCR analysis of *TXN* mRNA levels in shHSP90AB1 versus shCtrl CAL27/HSC3 cells (ns: not significant, one-way ANOVA).

(F) Western blot analysis of the indicated proteins in CAL27/HSC3 cells treated with cycloheximide (25 µg/ml) for the indicated time points (hours).

(G) Western blot analysis of the indicated proteins in CAL27/HSC3 cells treated with cycloheximide (25 µg/ml) in combination with NVP‑BEP800 (2 µM), MG132 (10 µM), CQ (50 µM), or the indicated combinations


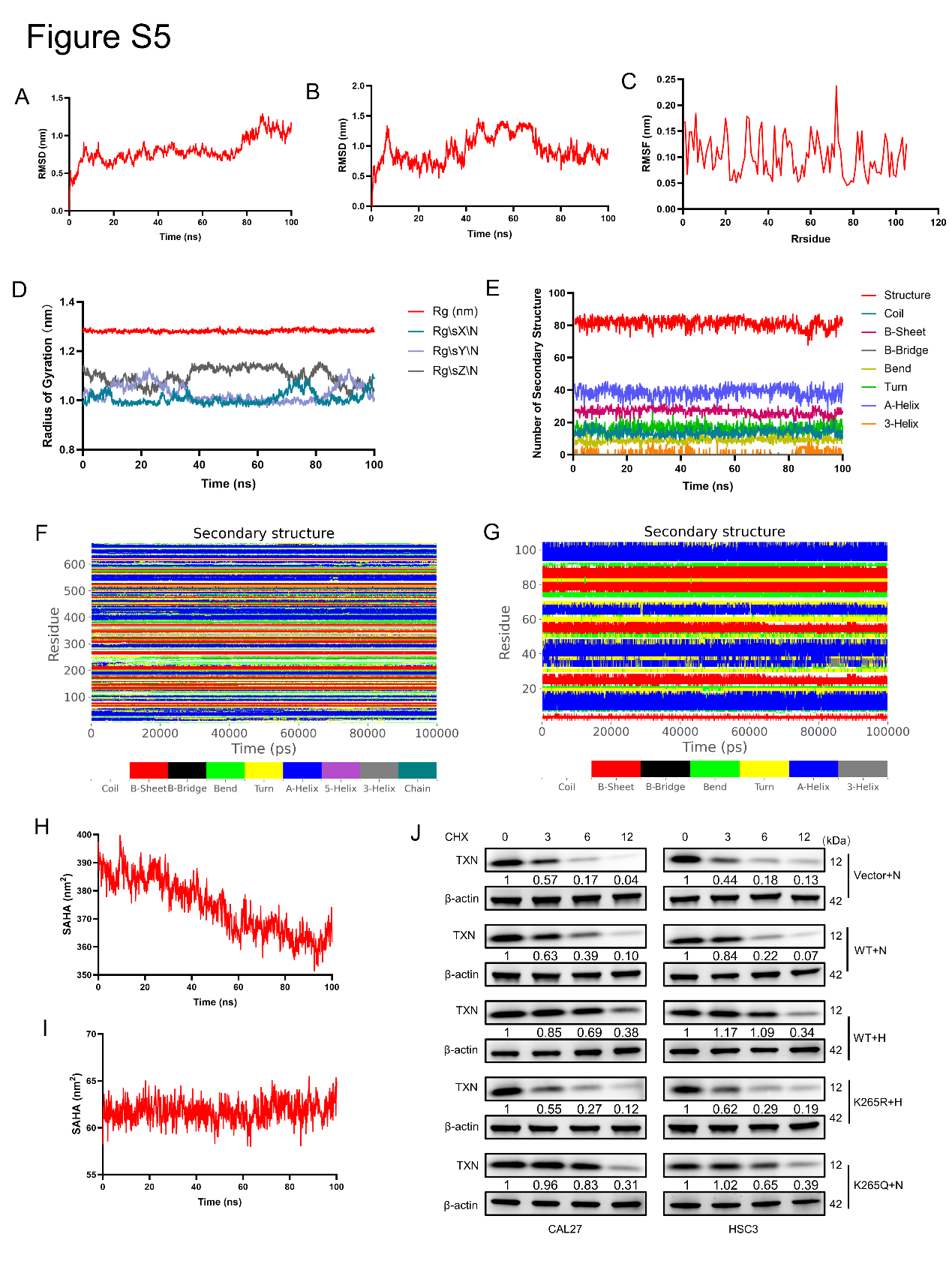


**Supplementary Figure 5: Extended molecular dynamics parameters and TXN stability analysis.**

(A-B) Root Mean Square Deviation trajectory of (A) HSP90AB1 and (B) TXN during molecular dynamics simulation.

(C) Root Mean Square Fluctuation per residue analysis of TXN.

(D) Radius of gyration measurements of TXN during simulation.

(E) Secondary structure composition analysis of TXN (DSSP method).

(F-G) Residue-level secondary structure assignment for (F) HSP90AB1 and (G) TXN.

(H-I) Solvent accessible surface area measurements of (H) HSP90AB1 and (I) TXN.

(J) Western blot analysis of the indicated proteins in CAL27 and HSC3 cells expressing different HSP90AB1 plasmids under indicatied conditions treated with cycloheximide (25 µg/ml) for the indicated time points (hour).


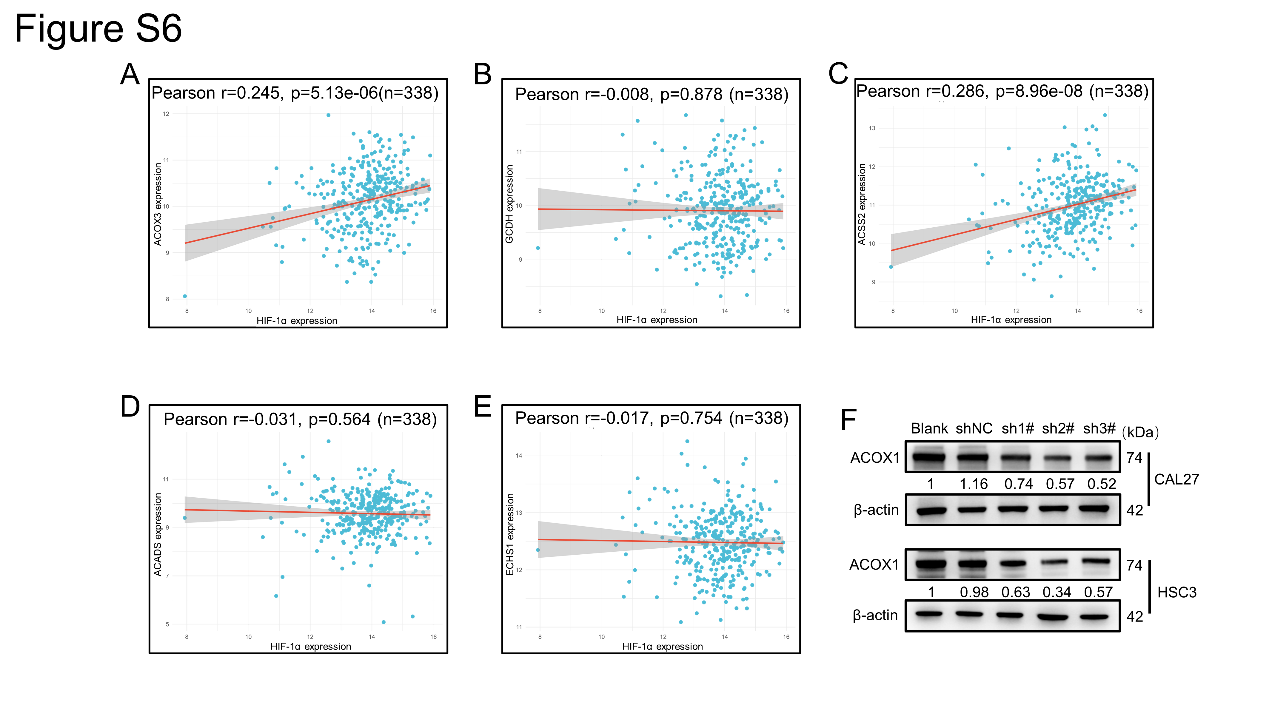


**Supplementary Figure 6: Transcriptional correlations between *HIF-1α* and crotonyl-CoA metabolic enzymes and ACOX1 knockdown validation**

(A-E) Scatter plot of *HIF-1α* versus (A) *ACOX3*, (B) *GCDH*, (C) *ACSS2*, (D) *ACADS* and (E) *ECHS1* mRNA expression levels in OSCC samples from TCGA cohort.

(F) Western blot analysis of ACOX1 protein levels in lentiviral shRNA-transduced CAL27 and HSC3 cells. β-actin: loading control.


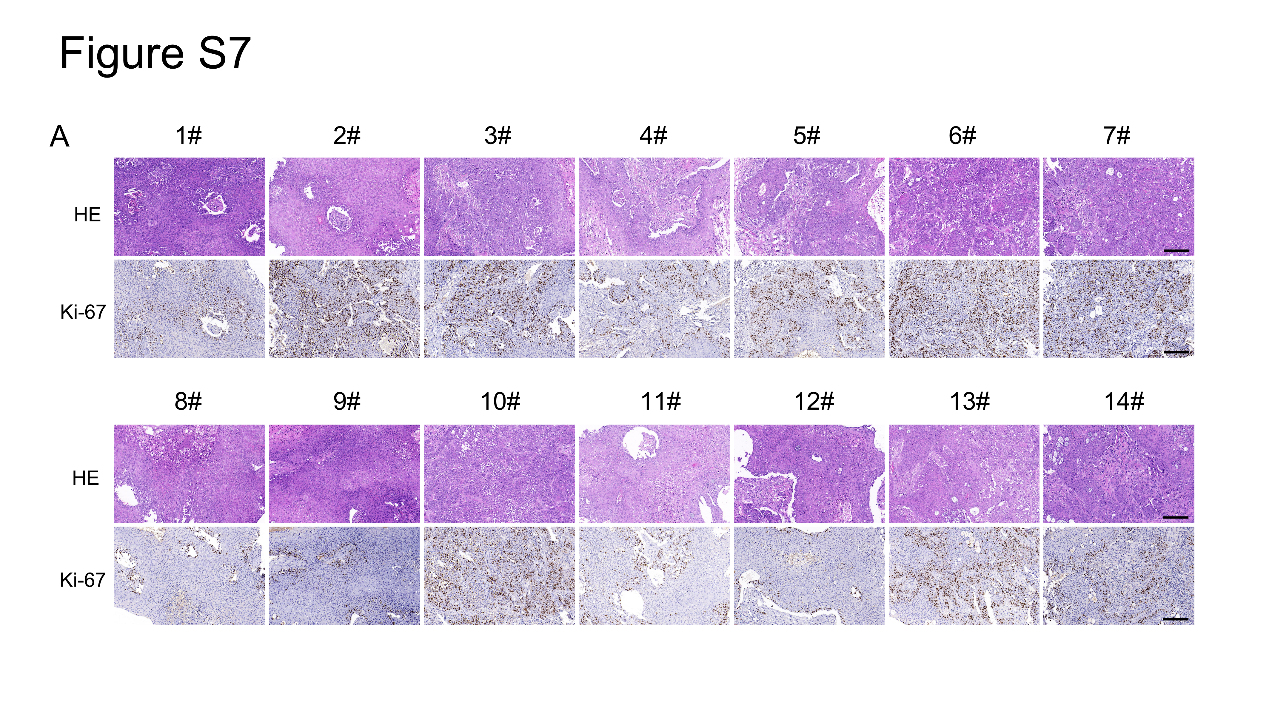
**Supplementary Figure 7: Representative H&E staining and Ki-67 IHC of tumor sections. (200X, scale bars, 100 µm)**


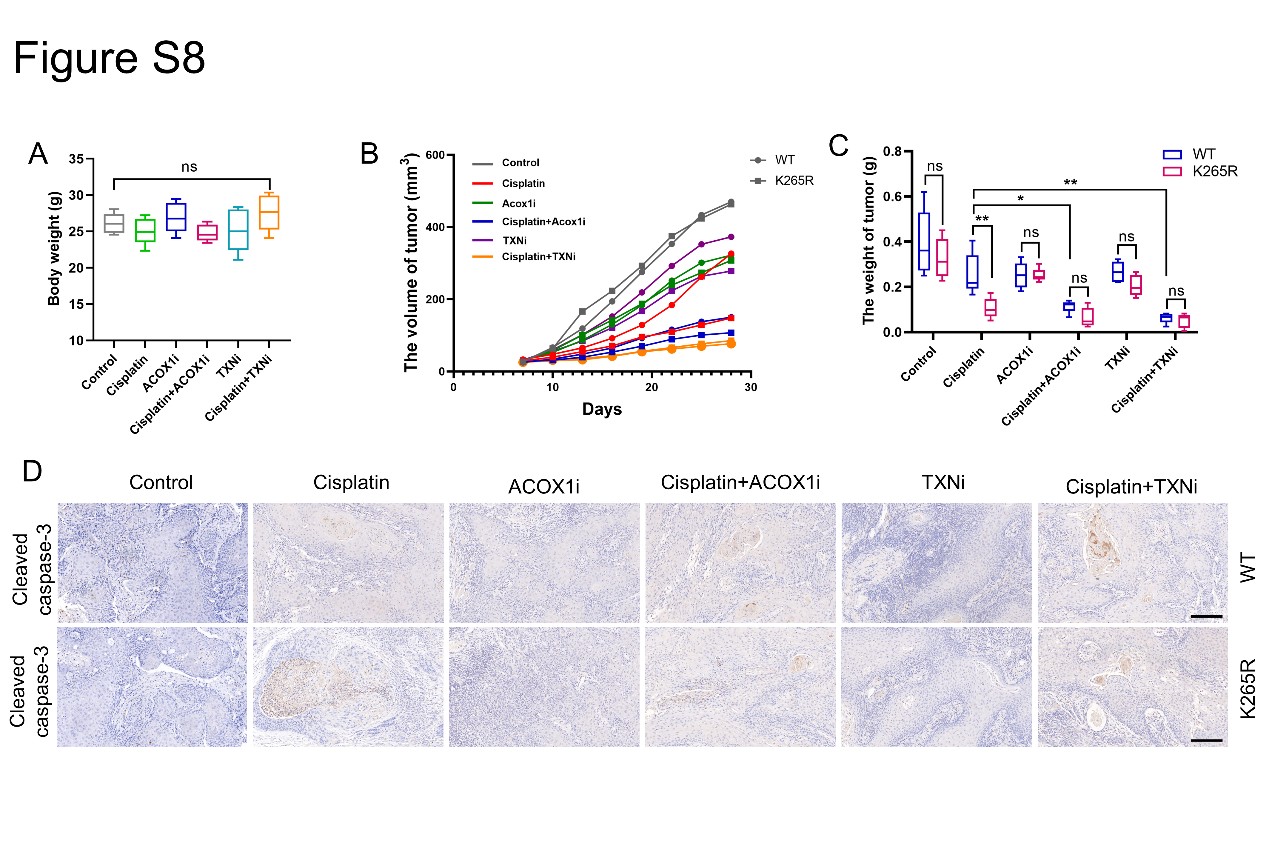


**Supplementary Figure 8: Safety and efficacy metrics of combination therapies**

(A) The body weights were compared among different groups. (ns: not significant, one-way ANOVA).

(B) Mean tumor volume over time for each group is shown (n=5 mice/group). Error bars are omitted for clarity due to the high number of groups.

(C) The tumor volumes were compared among different groups (*P<0.05, **P<0.01, ns: not significant; paired t-test and unpaired t-test).

(D) Representative cleaved caspase-3 IHC images of tumor sections (200X, scale bars, 100 µm).

**Supplementary Table 1. The sequences of primers used in this study**

| Primer | Sequence |
| --- | --- |
| *TXN* forward | 5’-GTAGTTGACTTCTCAGCCACGTG-3’ |
| *TXN* reverse | 5’-CTGACAGTCATCCACATCTACTTC-3’ |
| *ACOX1* forward | 5’- TGTCCTATTTGAACGACCTGCCCA -3’ |
| *ACOX1* reverse | 5’- AGGTTCCAAGCTACCTCCTTGCTT -3’ |
| *HSP90AB1* forward | 5’- TTGACATCATCCCCAACCCTC-3’ |
| *HSP90AB1* reverse | 5’-ACCAAACTGCCCAATCATGGA-3’ |
| *β-actin* forward | 5’-GCACCGTCAAGGCTGAGAAC-3’ |
| *β-actin* reverse | 5’-AGCACTGTGTTGGCGTACAG-3’ |
